# Going beyond the spacing effect: Does it matter how time on a task is distributed?

**DOI:** 10.1177/17470218221113933

**Published:** 2022-07-28

**Authors:** Dillon H Murphy, Robert A Bjork, Elizabeth L Bjork

**Affiliations:** Department of Psychology, University of California Los Angeles, Los Angeles, CA, USA

**Keywords:** Spacing effect, repetition effect, free recall, difficulty

## Abstract

We assessed the effects of removing some constraints that characterise traditional experiments on the effects of spaced, rather than massed, study opportunities. In five experiments—using lists of to-be-remembered words—we examined the effects of how total study time was distributed across multiple repetitions of a given to-be-remembered word. Overall, within a given list, recall profited from study time being distributed (e.g., four 1-s presentations or two 2-s presentations vs one 4-s presentation). Among the implications of these findings is that if students choose to engage in massed studying (by virtue of constraints on their study time or a failure to appreciate the benefits of spaced study sessions), then studying the information twice but for half the time may produce memory benefits in a single study session.

To achieve successful learning, students need to embrace, rather than avoid, certain *desirable difficulties* (E. L. Bjork & Bjork, 2014; R. A. Bjork, 1994) such as testing and generating rather than restudying (DeWinstanley & Bjork, 2004; Halamish & Bjork, 2011; Roediger & Karpicke, 2006), varying the environmental context when studying (Smith et al., 1978), interleaving rather than blocking practice (e.g., Kornell & Bjork, 2008), and spacing rather than massing study sessions (i.e., the spacing effect; R. A. Bjork & Allen, 1970; Cepeda et al., 2006; Greene, 2008; Karpicke & Bauernschmidt, 2011), which have all been shown to enhance learning outcomes. In the present research, we focused on whether the benefits of spacing might be realisable even when a student might be either unaware of the benefits of spacing or unwilling/unable to restudy after a substantial delay.

## Theoretical perspectives on how study time should be distributed

Across the long history of research on the effects of spaced versus massed opportunities to study various types of to-be-learned material, a variety of theoretical mechanisms have been proposed to account for the benefits of spacing (see Delaney et al., 2010; Hintzman, 1974, 1976; Maddox, 2016; Toppino & Gerbier, 2014, for reviews). Early research provided evidence for the attenuation of attention whereby attention declines more during massed presentations compared with spaced presentations (Melton, 1970; Shaughnessy et al., 1972; Underwood, 1969; but see Zimmerman, 1975). Other work proposed a consolidation account whereby long-term recall depends on a to-be-learned item’s representation in memory being consolidated and that massed repetition of an item does not provide enough time for the effects of a first study trial to be consolidated before a second study trial is presented (see R. A. Bjork & Allen, 1970, for a test of the consolidation idea).

Most modern accounts of the spacing effect attribute the benefits of distributed practice to increased encoding variability, avoiding deficient processing of information during a repetition, or more effective study-phase retrieval processes. The encoding variability theory (see Crowder, 1976; Delaney et al., 2010; Estes, 1955; Gerbier & Toppino, 2015; Glenberg, 1976; Johnston & Uhl, 1976; Melton, 1970) suggests that the accompanying contextual information of each to-be-remembered item varies over time and after multiple presentations, a greater variety of information has been paired with each spaced to-be-remembered item in comparison to the massed ones, resulting in the former having more (or stronger) retrieval paths (see Howard & Kahana, 2002; Murdock, 1997; Polyn et al., 2009; Raaijmakers, 2003; Raaijmakers & Shiffrin, 1981, for the role of contextual encoding in several memory models).

Next, the deficient-processing account of the spacing effect suggests that when studying is massed, the additional study time for a given piece of information becomes redundant such that little effective processing occurs. In contrast, when restudying is spaced, the quality of processing during the additional study time is increased (see Hintzman & Block, 1971, 1973; Hintzman et al., 1973; Johnston & Uhl, 1976; Magliero, 1983; Shaughnessy et al., 1972).

The study-phase retrieval theory posits that when an item is repeated for study, the recognition of that item triggers the retrieval of the prior study experience for that item (Hintzman & Block, 1973; Hintzman et al., 1975). This manifestation of retrieval-practice benefits (R. A. Bjork, 1988; Roediger & Butler, 2011; see also Balota et al., 2007a) becomes even greater/stronger when the eventual later successful retrieval of an item is more difficult or delayed (Appleton-Knapp et al., 2005; Greene, 1989; Thios & D’Agostino, 1976). Each of these accounts of the spacing effect has been supported by experimental findings, and such explanations are not mutually exclusive (see Benjamin & Tullis, 2010), which may be why the benefits of spacing are so prevalent and general.

## Altering presentation rate and the number of presentations

Although the spacing effect is robust (Cepeda et al., 2006; Donovan & Radosevich, 1999; Kim et al., 2019), changing an item’s presentation rate can alter the effectiveness of spacing. For example, faster presentation rates can reduce the benefits of distributed practice (see Wenger, 1979) or even reverse the spacing effect (Metcalfe & Kornell, 2003; but see Ariel et al., 2014; Pyc & Dunlosky, 2010). Thus, ample time to study and encode to-be-remembered information on each presentation may be crucial for harnessing the benefits of the spacing effect (see Toppino et al., 2009, for an investigation of discrepancies in previous work resulting from differences in encoding time across studies).

## How the distribution of study time might affect the inter-association of temporally contiguous materials

When retrieving information from long-term memory, items studied together tend to be recalled together and in the order in which they were studied as opposed to randomly, a property known as the *lag-recency effect* (Kahana, 1996; Sederberg et al., 2010; Spillers & Unsworth, 2011). Specifically, contextual features facilitate the recall of items presented near one another during encoding. For example, two words presented close together in time share a temporal context and the opportunity to be inter-associated semantically, so that the retrieving of one of them can lead to the retrieval of another. When recently recalled words recruit accompanying contextual features to assist the retrieval of words presented nearby in the encoding phase, this retrieval tendency is captured by lag conditional-response probabilities (lag-CRPs; Kahana, 1996; see Hintzman, 2015, for a critique, but see Healey et al., 2019, for a response).

Previous work has demonstrated that the lag-recency effect is associated with better memory performance (Sederberg et al., 2010; Spillers & Unsworth, 2011) such that learners can enhance recall by using temporal-contextual cues of recently recalled items to facilitate the retrieval of more items. In contrast, a learner who fails to use the temporal-contextual cues of recently recalled words as a retrieval cue for additional words may experience poorer total recall. Thus, increased study opportunities may strengthen the shared temporal-contextual cues between to-be-remembered items and increase the lag-recency effect, potentially providing evidence that the benefits of spacing and repeated studying occur when additional temporal-contextual information is used to guide (and enhance) the retrieval process.

## Does the optimal distribution of study time vary as a function of the difficulty of to-be-learned materials?

There is evidence suggesting that the learning of more difficult information benefits less from spaced studying (Metzler-Baddeley & Baddeley, 2009) than the learning of less difficult information. As such, difficult material may be better learned in a single, longer encoding session allowing for more elaborative encoding and deeper levels of processing (Craik & Tulving, 1975). Furthermore, the qualities of the to-be-learned materials and the individual learners may be related to ideal spacing conditions. For example, prior work suggests that the benefits of repetition may depend on the individual learner’s ability (e.g., Agarwal et al., 2017) and the difficulty of the intervening task between repetitions can have different effects depending on the learner’s working memory ability (e.g., Bui et al., 2013; Verkoeijen & Bouwmeester, 2008). In addition, massing might be advantageous when studying difficult information as participants might be distributing their practice of the different elements of complex stimuli; however, simpler materials may allow for more relearning even within a single encoding session. Moreover, because there is not a second study opportunity, massing may increase the probability that a learner adequately learns difficult information. Thus, another question is whether breaking apart a given total amount of study time to utilise spacing and repetition effects (see Greene, 1989; Hintzman, 1976; Hintzman & Block, 1971; Raaijmakers, 2003) may be more beneficial for the learning of less difficult information than for the learning of more difficult information.

## The present research

Some prior work has shown the benefits of repetition and spacing within a single encoding session. For example, in a continuous paired-associate task, Glenberg (1976) demonstrated that distributing study time across multiple presentations within a list can enhance memory (see also Delaney et al., 2010; Maddox, 2016; see Maddox & Balota, 2015; Peterson et al., 1963, for examinations of the spacing effect utilising different retention intervals). In the present research, we were interested in (1) the potential benefits of distributed practice within a single encoding session (using unrelated word lists and free recall tests), (2) how this effect differs as a function of the difficulty of to-be-learned words, (3) how distributed practice effects temporal-contiguity effects, and (4) how potential distributed practice benefits recall as a function of study order (fixed vs random order).

We presented participants with lists of words to remember for a later test and each word received the same amount of total study time, but we manipulated (within-subjects) how that study time was distributed within each list (i.e., fewer, but longer, study opportunities or more, but shorter, study opportunities). We expected that increased (but shorter) encoding opportunities within a given study session would lead to better subsequent free recall of the studied words, but that any such benefits might be reduced or eliminated for more difficult (i.e., more abstract/less concrete) words.

In part, this expectation follows from Paivio’s (1971, 2013) dual-coding theory, which assumes that concrete words activate perceptual as well as verbal memory and are easier to remember compared with more abstract words (see also Schwanenflugel et al., 1988). Another consideration is that effective encoding strategies—such as interactive imagery, sentence generation, and grouping—lead to enhanced recall compared with less effective strategies, such as passive reading and simple repetition (Hertzog et al., 1998; Richardson, 1998; Unsworth, 2016), and participants studying more concrete (easier) words may be better able to utilise effective encoding strategies resulting in better performance on a later memory test than participants studying more abstract words. In addition, participants may only have time to engage in imagery, sentence generation, and other recall-enhancing activities when the to-be-remembered words are presented for longer study durations versus shorter, but more frequent, study opportunities (i.e., presentations for only 1 or 2 s at a time). As a result, repeated studying may only be beneficial for easier-to-remember words or when learners have enough study time to utilise elaborative encoding strategies for each item.

## Experiment 1a

In Experiment 1a, participants studied six lists of words, each of which contained 20 words. In two lists the words were presented once at a 4-s rate, in two lists the words were presented once at a 2-s rate and then again at a 2-s rate with the words in the same order, and in the remaining two lists the words were presented four times at a 1-s rate with the 20 words shown in the same order each time through the list. Thus, the total study time per word was kept constant across the three conditions (4 s). The order of the lists with each study schedule was counterbalanced, and the to-be-remembered words were either highly concrete (easier to remember) or more abstract (more difficult to remember).

### Method

#### Participants

After exclusions, participants were 86 undergraduate students (*M_age_* = 19.85, *SD_age_* = 1.43; 59 female, 27 male; 49 Asian/Pacific Islander, 1 Black, 18 Hispanic, 12 White, 6 other/unknown) recruited from the University of California Los Angeles (UCLA) Human Subjects Pool. Participants were tested online and received course credit for their participation. Participants were excluded from analysis if they admitted to cheating (e.g., writing down answers) in a post-task questionnaire (they were told they would still receive credit if they cheated). This exclusion process resulted in one exclusion. A sensitivity analysis based on the obtained sample indicated that for a 2 (word difficulty: easy, hard) × 3 (study schedule: 1 s × 4, 2 s × 2, 4 s × 1) mixed analysis of variance (ANOVA), assuming alpha = .05, power = .80, and a high correlation (*r* = .63) between repeated measures, the smallest effect (recall as a function of study schedule) the design could reliably detect is $\eta_{p}^{2}$ = .02 which is larger than most effects reported in memory research (see Morris & Fritz, 2013).

#### Materials

All studied words were nouns that contained four letters and participants either studied lists containing more concrete words (i.e., easier to remember; *n* = 44) or more abstract words (i.e., more difficult to remember; *n* = 42). Words were classified according to the English Lexicon Project website (Balota et al., 2007b) and word lists were formed by randomly sampling unique sets of 20 words from a pool of 303 (177 easy words and 126 hard words). Thus, each participant received different lists of words with a different combination of words in each list, and each word could appear on a list with any of the different study schedules.

For participants presented with easier words to remember, on the log-transformed Hyperspace Analogue to Language (HAL) frequency scale (with lower values indicating lower frequency in the English language and higher values indicating higher frequency), words ranged from 5.48 to 12.88 and averaged a score of 9.63 (*SD* = 1.44). In terms of concreteness (with lower values indicating lower concreteness and higher values indicating higher concreteness), these words ranged from 4.26 to 5.00 and averaged a score of 4.74 (*SD* = 0.20). For participants presented with harder-to-remember words, frequency levels ranged from 7.43 to 14.35 and averaged a score of 10.70 (*SD* = 1.16), and their concreteness levels ranged from 1.25 to 4.24 and averaged a score of 3.26 (*SD* = 0.71). Words we classified as “hard” were significantly more concrete than words we classified as “easy,” *t*(301) = 26.34, *p* < .001, *d* = 3.07, BF_10_ > 100, but were also significantly less frequent than “easy” words, *t*(301) = 6.89, *p* < .001, *d* = .80, BF_10_ > 100.^1^

#### Procedure

**Figure 1.**
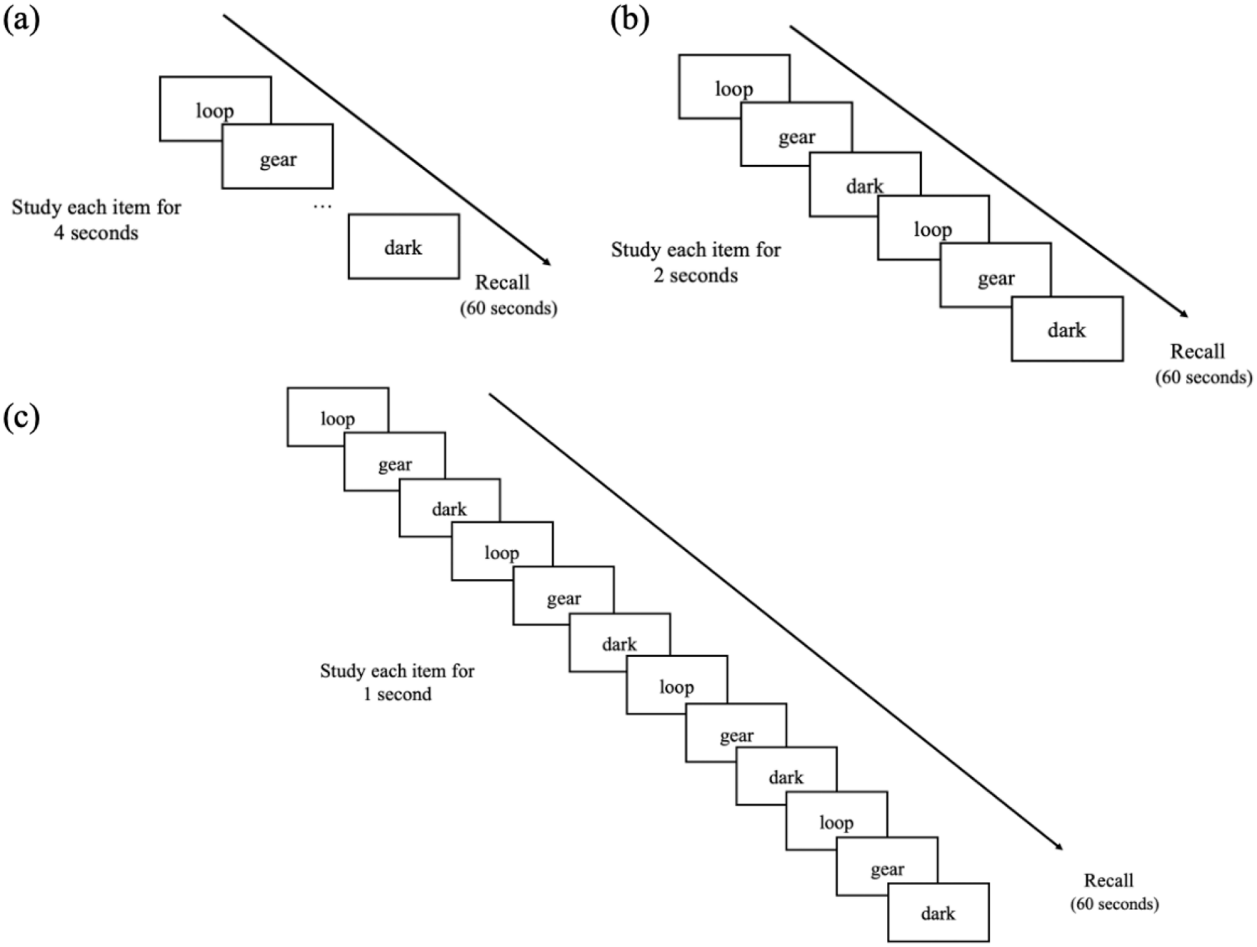
Example of (a) a list with each word presented once for 4 s, (b) a list with each word presented twice for 2 s each, and (c) a list with each word presented four times for 1 s each in Experiment 1a.

Participants were told that they would be presented with lists of words with each list containing 20 words and that their task was to remember the words for a later test. Participants were presented with six lists in total and on each list, participants either viewed each word once for 4 s (two lists), twice for 2 s (two lists), or four times for 1 s (two lists; see Figure 1). List order was counterbalanced, but study conditions occurred in blocks (i.e., the two lists where words were studied once for 4 s occurred consecutively). On lists where participants viewed the words more than once, the order of words was the same across cycles throughout the list (i.e., words 1–20 were presented once, then again in the same order). After the presentation of all 20 words, participants were given a 1 min immediate free recall test in which—in an on-screen text box—they recalled as many words as they could from the just-studied list in any order they wished. Immediately following the recall period, participants were informed of the number of correctly recalled words for that list but were not given feedback about specific words.

Following the test of the final to-be-remembered list, participants reported what encoding strategies (if any) they had used to remember the words using a check-off list of possible strategies. Specifically, participants indicated whether they simply read each word as it appeared, repeated the words as much as possible, developed rhymes for the words, used sentences to link the words together, developed mental images of the words, grouped the words in a meaningful way, or did not use any strategies. Participants could select some, all, or none of the suggestions to indicate which strategies they used.

### Results

**Figure 2.**
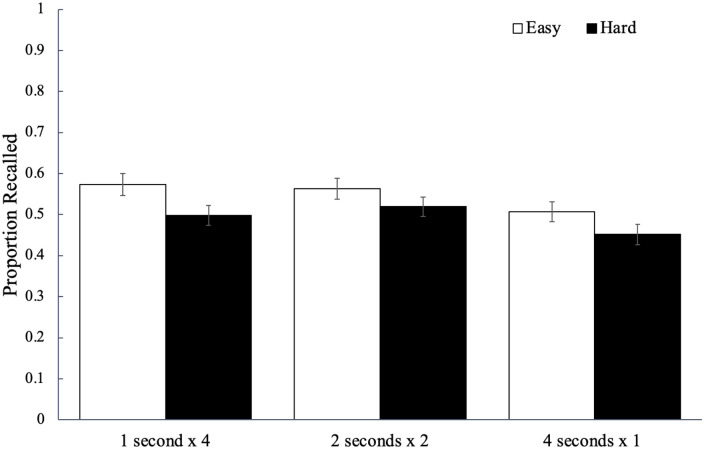
The average proportion of words correctly recalled as a function of word difficulty and how a fixed study time was distributed across presentations of a given word in Experiment 1a. Error bars reflect the standard error of the mean.

Recall performance for each study schedule as a function of word difficulty is shown in Figure 2 and to analyse potential differences, we conducted a 2 (word difficulty: easy, hard) × 3 (study schedule: 1 s × 4, 2 s × 2, 4 s × 1) mixed ANOVA. To examine the strength of the evidence for each effect, we also computed a Bayes factor (a ratio of the marginal likelihood of the null model and a model suggesting group differences) compared with a null model using JASP (Love et al., 2019). We provide BF_01_ when inferential statistics favour the null hypothesis (which would be supported by a large BF_01_) and BF_10_ when inferential statistics favour the alternative hypothesis (which would be supported by a large BF_10_; for more information on interpreting Bayes factors, see Kass & Raftery, 1995). Results did not reveal a significant effect of word difficulty, *F*(1, 84) = 3.64, *p* = .060, $\eta_{p}^{2}$ = .04, BF_01_ = .82,^2^ with participants recalling a similar proportion of easy words (*M* = 0.55, *SD* = 0.14) as hard words (*M* = 0.49, *SD* = 0.14). However, there was a significant main effect of study schedule, *F*(2, 168) = 9.91, *p* < .001, $\eta_{p}^{2}$ = .11, BF_10_ > 100, such that words studied four times for 1 s (*M* *=* 0.54, *SD* *=* 0.17) were recalled better than the words studied once for 4 s (*M* *=* 0.48, *SD* = 0.16, *p*_bonf_ = .003, *d* = 0.37) but not better than the words studied twice for 2 s (*M* = 0.54, *SD* = 0.16, *p*_bonf_ > .999, *d* = 0.04); additionally, recall for the words studied twice for 2 s was greater than that for the words studied once for 4 s (*p*_bonf_ < .001, *d* = 0.46). Word difficulty did not interact with study schedule, *F*(2, 168) = 0.52, *p* = .596, $\eta_{p}^{2}$ = .01, BF_01_ = 9.67.

Although results indicate that studying a word multiple times but for a shorter duration can enhance memory, the retention interval differs between these study schedules. For example, when studying each word once for 4 s, the time until the recall test is 76 s for the first word, whereas when the first word is studied for the final time when studying each word four times for 1 s each, the time until the recall test is 19 s. To examine the potential benefits of distributing study time while controlling for retention interval, we conducted a logistic multilevel model (MLM) where we treated the data as hierarchical or clustered (i.e., multilevel) and items nested within individual participants. In this analysis, the regression coefficients are given as logit units (i.e., the log odds of correct recall). We report exponential betas (e^B^), and their 95% confidence intervals, which give the coefficient as an odds ratio (i.e., the odds of correctly recalling a word divided by the odds of not recalling a word). Thus, e^B^ can be interpreted as the extent to which the odds of correctly recalling a word changed. Specifically, values greater than 1 represent an increased likelihood of recall while values less than 1 represent a decreased likelihood of recall.

To examine recall as a function of the number of word presentations while controlling for retention interval (for the final presentation of each word, we calculated the time remaining until the beginning of the recall test), we conducted a logistic MLM with item-level recall modelled as a function of number of word presentations and retention interval. Results revealed that, when controlling for retention intervals, the number of word presentations significantly predicted recall, e^B^ = 1.11, confidence interval (CI) = [1.07, 1.16], *z* = 5.25, *p* < .001, such that distributed practice enhanced memory.

As previously mentioned, CRPs measure how memory performance is affected by accompanying temporal and contextual information and measure how individuals transition between responses during recall. The CRP for each recall transition is computed by summing the number of times the transition of a certain lag occurred divided by the number of times that transition could have occurred. Lag is the ordinal distance between successively recalled words (i.e., the lag between output position 5 and 7 would be 2) and the sign of the lag indicates the direction of recall (positive values indicate forward and negative values indicate backward). Thus, CRPs illustrate the probability that an item from serial position *i* + lag is recalled immediately following an item from serial position *i*. For example, if an individual recalls an item presented in serial position 10, the CRP for a lag of 1 would be the probability that the item in serial position 10 is recalled immediately after the item in serial position 9 or 11 (as opposed to 4 or 5, for example).

**Figure 3.**
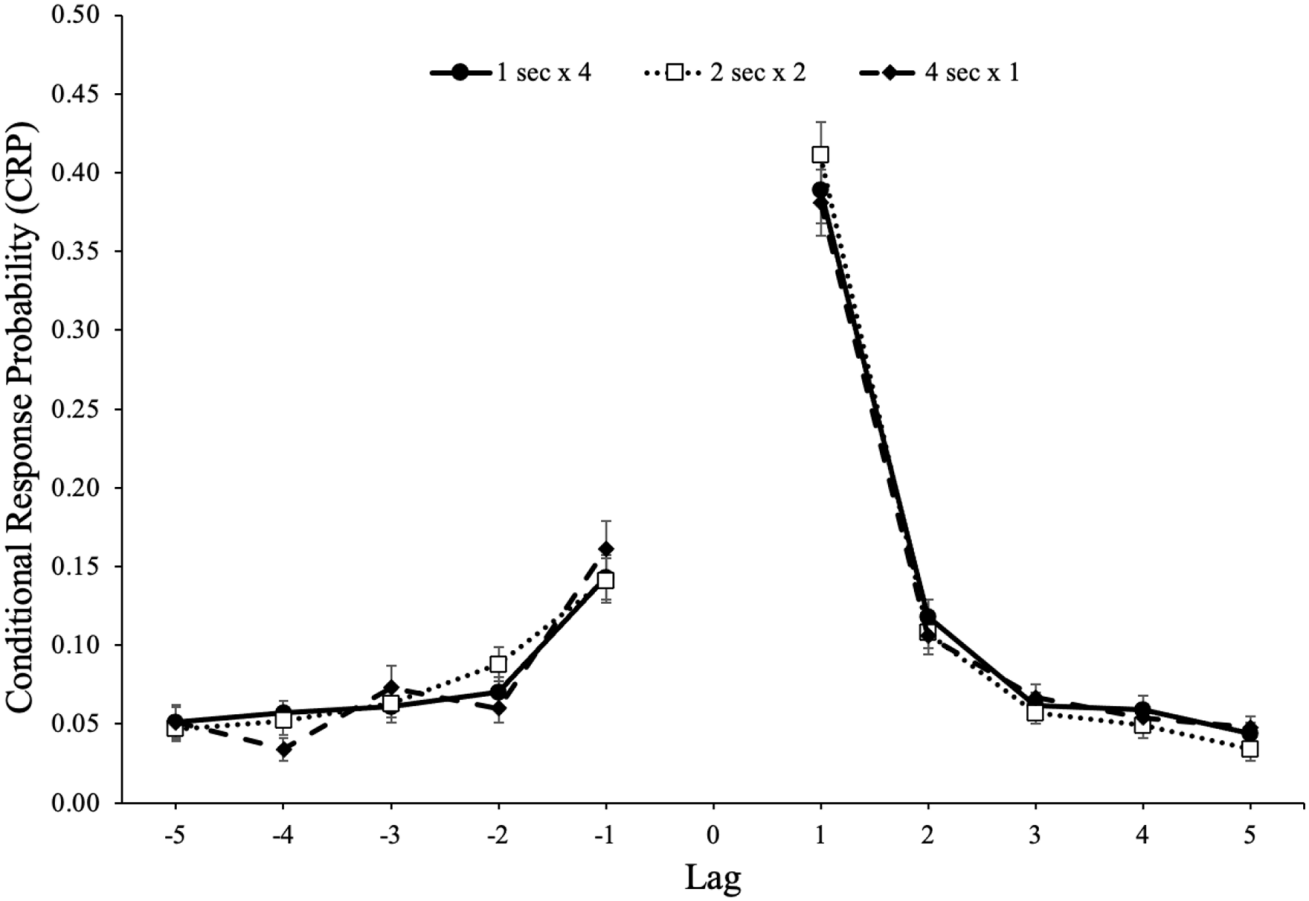
Conditional-response probability (CRP) functions for forward and backward transitions as a function of lag and study schedule in Experiment 1a. Error bars reflect the standard error of the mean.

The probability of recalling an item from serial position *x* followed by an item from serial position *y* is shown in Figure 3. A 5 (lag: 1–5) × 2 (direction: forward vs backward) × 2 (study schedule: 1 s × 4, 2 s × 2, 4 s × 1) repeated-measures ANOVA^3^ revealed a forward preference for the direction of transitions, *F*(1, 85) = 146.87, *p* < .001, $\eta_{p}^{2}$ = .63, BF_10_ > 100, and strong adjacency effects, *F*(4, 340) = 300.84, *p* < .001, $\eta_{p}^{2}$ = .78, BF_10_ > 100. In addition, lag interacted with direction, *F*(4, 340) = 89.08, *p* < .001, $\eta_{p}^{2}$ = .51, BF_10_ > 100, such that participants demonstrated a stronger preference for words in the forward direction of 1 lag. However, there was not a main effect of study schedule, *F*(2, 170) = 0.15, *p* = .861, $\eta_{p}^{2}$ < .01, BF_01_ > 100, such that participants showed similar lag-recency effects whether studying each item once for 4 s, twice for 2 s, or four times for 1 s. Furthermore, study schedule did not interact with direction, *F*(2, 170) = 0.10, *p* = .902, $\eta_{p}^{2}$ < .01, BF_01_ = 99.54, or lag, *F*(8, 680) = 0.74, *p* = .659, $\eta_{p}^{2}$ = .01, BF_01_ > 100, and there was not a three-way interaction between direction, lag, and study schedule, *F*(8, 680) = 0.97, *p* = .463, $\eta_{p}^{2}$ = .01, BF_01_ > 100.

**Figure 4.**
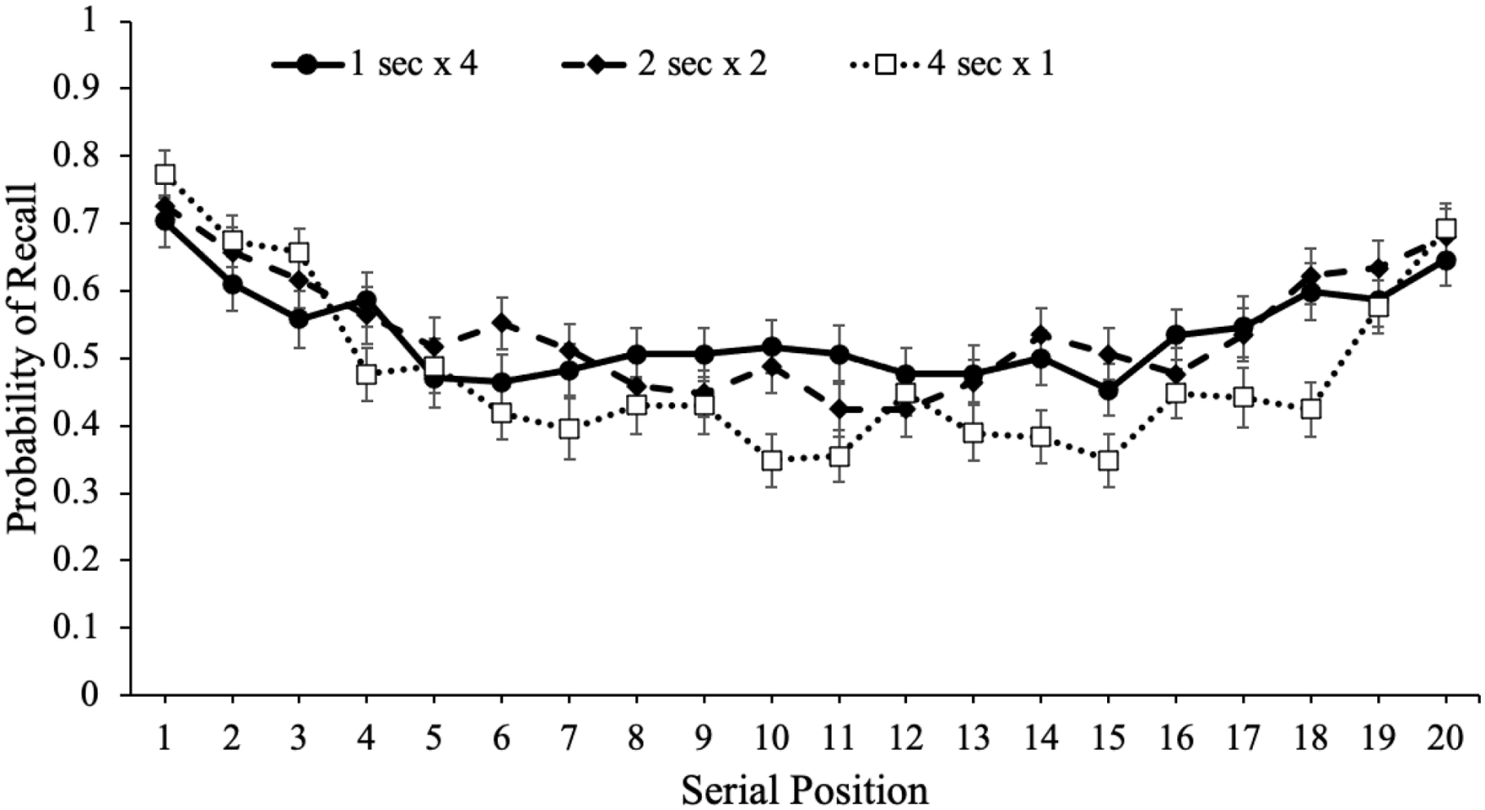
Free recall probability as a function of study schedule and serial position in Experiment 1a. Error bars reflect the standard error of the mean.

To potentially account for the recall advantage for words presented multiple times within a given list, we also investigated serial position effects for each study schedule, which are shown in Figure 4. Serial position effects refer to an increased probability of recall for words presented in the beginning (primacy effect) and end (recency effect) of a list compared with words in the middle of the list (Glanzer & Cunitz, 1966; Murdock, 1962; Waugh & Norman, 1965). Primacy effects are largely attributable to rehearsal such that during a given item’s presentation, participants also typically rehearse previously presented words leading to more rehearsal and better recall for primacy items (see Fischler et al., 1970; Rundus, 1971; Rundus & Atkinson, 1970). In terms of the recency effect, when the presentation of the last item immediately precedes a recall test, participants often dump the last few presented items from working memory stores resulting in enhanced recall for these items (Crowder, 1969). However, if a delay follows the study phase, recall for recency items tends to be similar to—or even worse than—items presented in the middle of the list (R. A. Bjork, 1975; Craik, 1970; Glanzer & Cunitz, 1966; Howard & Kahana, 1999; Waugh & Norman, 1965).

To examine recall as a function of serial position as well as the number of word presentations, we conducted a logistic MLM with item-level recall modelled as a function of serial position and number of word presentations which revealed that serial position significantly predicted recall, e^B^ = 0.99, CI = [0.98, 0.99], *z* = –3.60, *p* < .001, such that primacy and recency items were better recalled than middle items. In addition, number of word presentations significantly predicted recall, e^B^ = 1.07, CI = [1.01, 1.11], *z* = 4.16, *p* < .001, and serial position interacted with the number of word presentations, e^B^ = 1.01, CI = [1.00, 1.01], *z* = 2.57, *p* = .010, such that serial position was a stronger predictor of recall when participants were presented with words fewer times.

To supplement these findings, we also computed quadratic regressions with serial position predicting recall for each study schedule. As shown in Table 1, quadratic models significantly predicted recall such that there were serial position effects in participants’ recall whereby primacy and recency items were recalled better than items in the middle of the list. However, the more times participants were presented with each word, the flatter the serial position curve.^4^ Thus, words presented in the middle of the list appeared to benefit the most from spaced repetitions.

**Table 1.** Quadratic regressions with recall predicted by serial position for each study schedule in Experiment 1a.

Study schedule	*R* ^2^	*b*1	*b*2	*F*	*p*
1 s × 4	.013	−.041	.002	23.26	<.001
2 s × 2	.027	−.059	.003	47.59	<.001
4 s × 1	.052	−.083	.004	93.68	<.001

### Discussion

To summarise, the pattern of results obtained in Experiment 1a revealed a recall advantage for words studied multiple times compared to once (despite no differences in total study time), but this finding did not differ according to item difficulty and there were no differences in the lag-recency effect as a function of study schedule. Experiment 1b was designed to examine whether aspects of the results of Experiment 1a might be attributable to participants being able to immediately recall the words in each list after the presentation of the last word in the list.

## Experiment 1b

In Experiment 1b, participants completed a similar task as in Experiment 1a, but with a 30-s distraction task occurring between the presentation of each list and the subsequent cue to free recall the list. Similar to Experiment 1a, we expected increased (although shorter) study opportunities to produce improved memory performance overall, but to a greater extent for the more concrete words.

### Method

#### Participants

After exclusions, participants were 91 undergraduate students (*M_age_* = 20.04, *SD_age_* = 1.86; 62 female, 28 male, 1 other; 37 Asian/Pacific Islander, 4 Black, 18 Hispanic, 25 White, 7 other/unknown) recruited from the UCLA Human Subjects Pool. Participants were tested online and received course credit for their participation. Participants were excluded from analysis if they admitted to cheating (e.g., writing down answers) in a post-task questionnaire (they were told they would still receive credit if they had cheated). This exclusion process resulted in two exclusions. A sensitivity analysis based on the obtained sample indicated that for a 2 (word difficulty: easy, hard) × 3 (study schedule: 1 s × 4, 2 s × 2, 4 s × 1) mixed ANOVA, assuming alpha = .05, power = .80, and a high correlation (*r* = .63) between repeated measures, the smallest effect (recall as a function of study schedule) the design could reliably detect is $\eta_{p}^{2}$ = .01.

#### Materials and procedure

The task in Experiment 1b was similar to the task in Experiment 1a except that instead of completing each free recall test immediately after the study phase, participants first completed a 30-s distraction task that required them to rearrange the digits of several three-digit numbers in descending order (e.g., 123 would be rearranged to 321; adapted from Rohrer & Wixted, 1994; Unsworth, 2007). Participants were given 3 s to view each of the 10 three-digit numbers and subsequently rearrange the digits. Similar to Experiment 1a, participants either studied lists containing more concrete words (i.e., easier words to remember; *n* = 46) or less concrete words (i.e., more difficult words to remember; *n* = 45).

### Results

**Figure 5.**
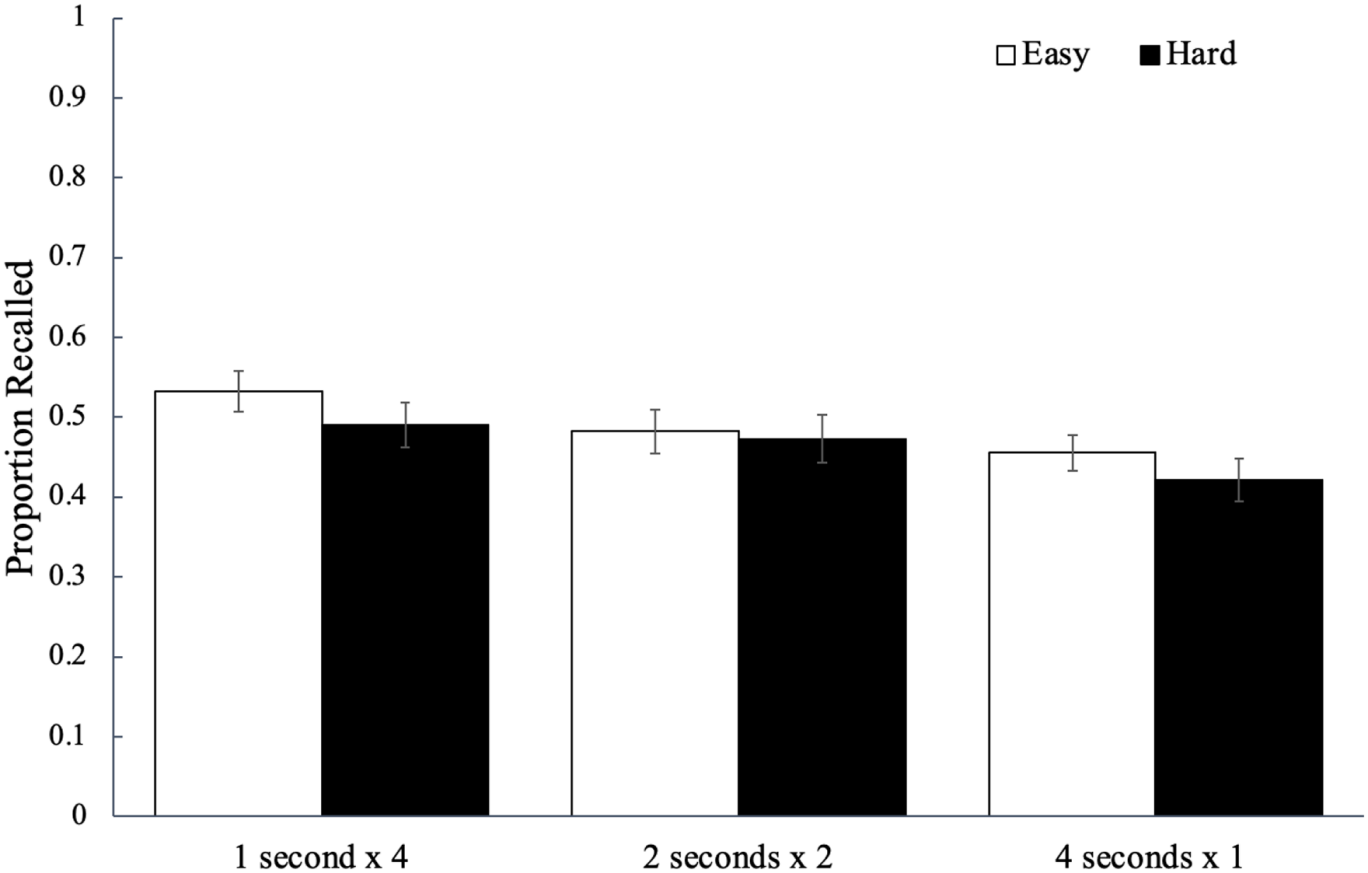
The average proportion of words correctly recalled as a function of word difficulty and how a fixed study time was distributed across presentations of a given word in Experiment 1b. Error bars reflect the standard error of the mean.

Recall performance for each study schedule as a function of word difficulty is shown in Figure 5. A 2 (word difficulty: easy, hard) × 3 (study schedule: 1 s × 4, 2 s × 2, 4 s × 1) mixed ANOVA did not reveal a significant main effect of word difficulty, *F*(1, 89) = 0.76, *p* = .387, $\eta_{p}^{2}$ = .01, BF_01_ = 2.62, such that participants recalled a similar proportion of easy words (*M* = 0.49, *SD* = 0.15) as hard words (*M* = 0.46, *SD* = 0.16). However, there was a main effect of study schedule, *F*(2, 178) = 10.07, *p* < .001, $\eta_{p}^{2}$ = .10, BF_10_ > 100, such that words studied four times for 1 s (*M* *=* 0.51, *SD* *=* 0.18) were recalled better than the words studied once for 4 s (*M* *=* 0.44, *SD* = 0.16, *p*_bonf_ < .001, *d* = 0.48) but not the words studied twice for 2 s (*M* = 0.48, *SD* = 0.19, *p*_bonf_ = .156, *d* = 0.21); additionally, recall for the words studied twice for 2 s was significantly greater than recall for the words studied once for 4 s (*p*_bonf_ = .040, *d* = 0.27). Word difficulty did not interact with study schedule, *F*(2, 178) = 0.55, *p* = .579, $\eta_{p}^{2}$ = .01, BF_01_ = 8.30.

To examine recall as a function of the number of word presentations while controlling for retention interval, we conducted a logistic MLM with item-level recall modelled as a function of number of word presentations and retention interval. Results revealed that, when controlling for retention intervals, the number of word presentations significantly predicted recall, e^B^ = 1.24, CI = [1.19, 1.29], *z* = 10.73, *p* < .001, such that distributed practice enhanced memory.

**Figure 6.**
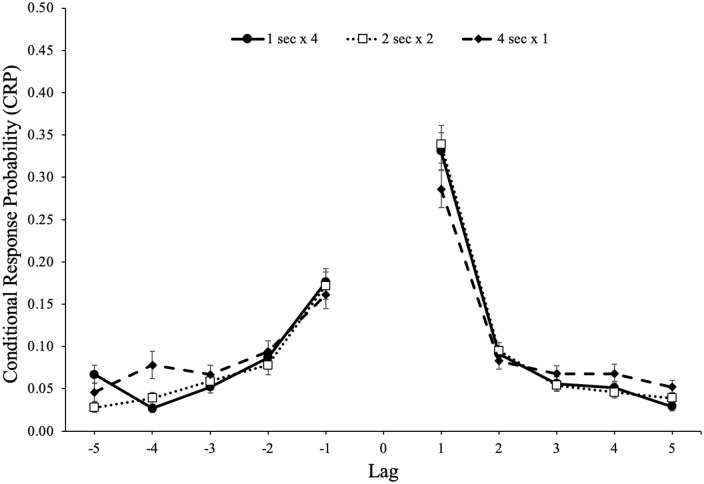
Conditional-response probability (CRP) functions for forward and backward transitions as a function of lag and study schedule in Experiment 1b. Error bars reflect the standard error of the mean.

CRP functions for forward and backward transitions as a function of lag and study schedule are shown in Figure 6. A 5 (lag: 1–5) × 2 (direction: forward vs backward) × 2 (study schedule: 1 s × 4, 2 s × 2, 4 s × 1) repeated-measures ANOVA revealed a forward preference for the direction of transitions, *F*(1, 90) = 38.21, *p* < .001, $\eta_{p}^{2}$ = .30, BF_10_ > 100, and strong adjacency effects, *F*(4, 360) = 193.78, *p* < .001, $\eta_{p}^{2}$ = .68, BF_10_ > 100. In addition, lag significantly interacted with direction, *F*(4, 360) = 35.10, *p* < .001, $\eta_{p}^{2}$ = .28, BF_10_ > 100, such that participants demonstrated a stronger preference for words in the forward direction of 1 lag. However, there was not a significant main effect of study schedule, *F*(2, 180) = 0.93, *p* = .395, $\eta_{p}^{2}$ = .01, BF_01_ > 100, such that participants showed similar lag-recency effects whether studying each item once for 4 s, twice for 2 s, or four times for 1 s. Moreover, study schedule did not interact with direction, *F*(2, 180) = 2.06, *p* = .130, $\eta_{p}^{2}$ = .02, BF_01_ = 47.48, but there was an interaction between study schedule and lag, *F*(8, 720) = 3.23, *p* = .001, $\eta_{p}^{2}$ = .04, BF_10_ = 0.08, although there were no significant pairwise comparisons of interest (and Bayes factor does not support this interaction). Furthermore, there was not a three-way interaction between direction, lag, and study schedule, *F*(8, 720) = 1.23, *p* = .278, $\eta_{p}^{2}$ = .01, BF_01_ > 100.

**Figure 7.**
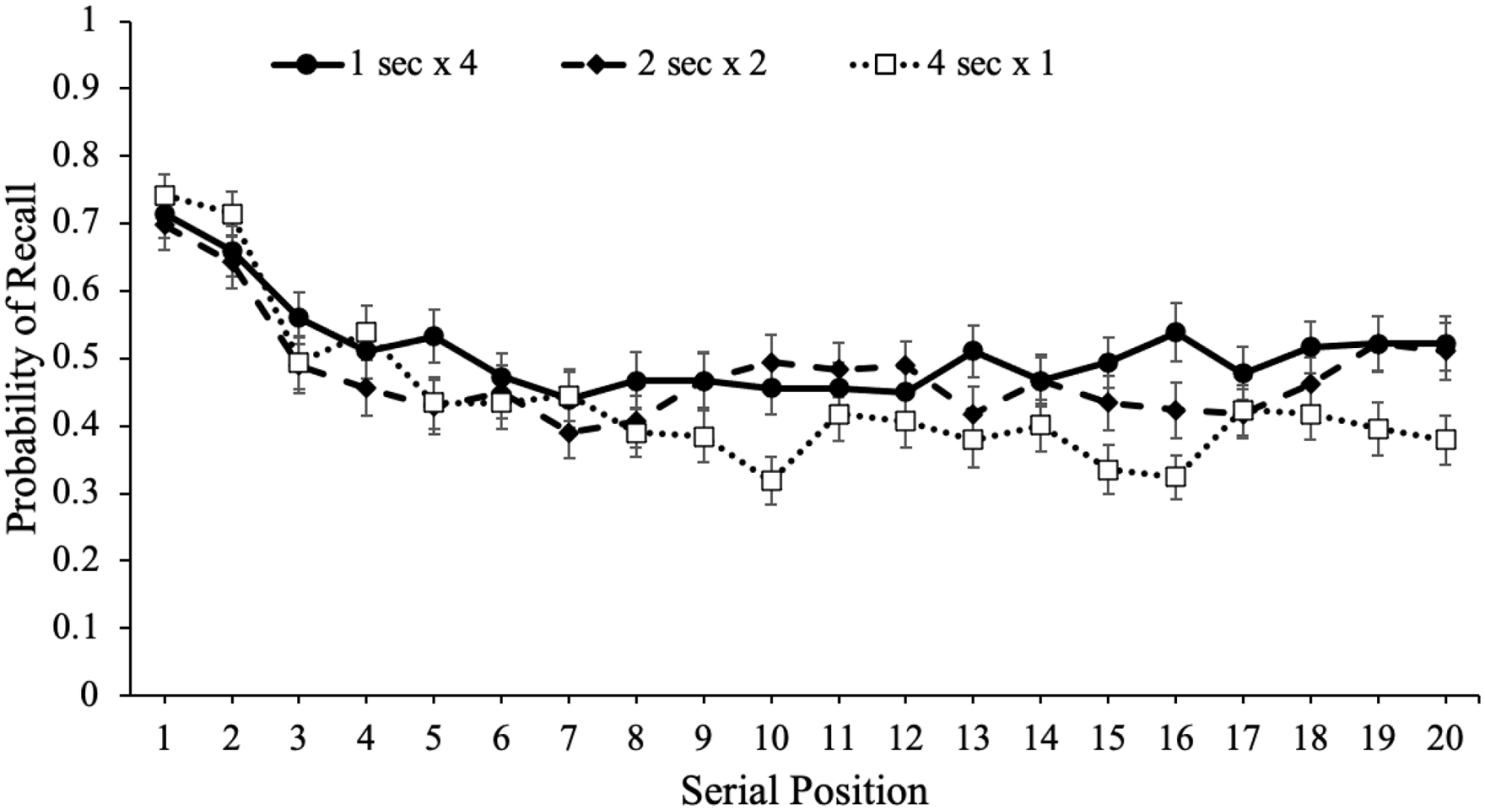
Free recall probability as a function of study schedule and serial position in Experiment 1b. Error bars reflect the standard error of the mean.

Next, we again investigated serial position effects for each study schedule, which are shown in Figure 7. A logistic MLM with item-level recall modelled as a function of serial position and the number of word which presentations revealed that serial position significantly predicted recall, e^B^ = 0.97, CI = [0.96, 0.97], *z* = –9.46, *p* < .001, such that primacy items were better recalled than middle and recency items. In addition, the number of word presentations significantly predicted recall, e^B^ = 1.11, CI = [1.08, 1.15], *z* = 6.43, *p* < .001, and serial position interacted with the number of word presentations, e^B^ = 1.01, CI = [1.01, 1.02], *z* = 3.70, *p* < .001, such that serial position was a stronger predictor of recall when participants were presented with items once or twice.

To supplement these findings, we again computed quadratic regressions with serial position predicting recall for each study schedule. As shown in Table 2, quadratic models significantly predicted recall such that there were serial position effects in participants’ recall whereby primacy items were recalled better than items in the middle and at the end of the list. However, these models also demonstrated that when participants were presented with each word four times, the serial position curve flattened.

**Table 2.** Quadratic regressions with recall predicted by serial position for each study schedule in Experiment 1b.

Study schedule	*R*2	*b*1	*b*2	*F*	*p*
1 s × 4	.014	−.041	.002	25.25	<.001
2 s × 2	.012	−.038	.002	21.39	<.001
4 s × 1	.039	−.057	.002	73.24	<.001

### Discussion

Similar to Experiment 1a, studying an item multiple times but for shorter durations in a massed encoding phase resulted in better memory performance than studying each item a single time for a longer duration, even after a delay (and regardless of item difficulty). The lag-recency effect, however, again did not differ according to how many times an item was studied, but serial position effects were reduced when participants were presented with each word four times. Thus, the pattern of results observed in Experiment 1b is consistent with Experiment 1a and illustrates that studying information multiple times (non-sequentially) for shorter durations results in better memory performance than studying something a single time for a longer duration.

## Experiment 2a

In Experiment 1, the lists where participants studied each item once for 4 s may have confounded the effects of spacing and repetition. Specifically, without a massed, individual item-level repetition comparison (studying a given item four times for 1 s but in immediate succession), it is unclear whether the benefits observed in Experiment 1 are attributable to the number of repetitions or the distribution of study time. In Experiment 2, we included a massed equivalent of the 4 s × 1 and the 2 s × 2 conditions by repeating the words in adjacent fashion (i.e., 4 immediate repetitions of the same word for 1 s each or 2 immediate repetitions of the same word for 1 s each then 2 immediate repetitions of the same word for 1 s each later in the list, respectively) to allow a comparison of spacing and massing but controlling for the number of item presentations.

### Method

#### Participants

After exclusions, participants were 96 undergraduate students (*M_age_* = 20.30, *SD_age_* = 3.33; 81 female, 15 male; 48 Asian/Pacific Islander, 3 Black, 10 Hispanic, 25 White, 10 other/unknown) recruited from the UCLA Human Subjects Pool. Participants were tested online and received course credit for their participation. Participants were excluded from analysis if they admitted to cheating (e.g., writing down answers) in a post-task questionnaire (they were told they would still receive credit if they cheated). This exclusion process resulted in one exclusion. A sensitivity analysis based on the obtained sample indicated that for a 2 (word difficulty: easy, hard) × 3 (study schedule: 1 s × 4, 2 s × 2, 4 s × 1) mixed ANOVA, assuming alpha = .05, power = .80, and a high correlation (*r* = .74) between repeated measures, the smallest effect (recall as a function of study schedule) the design could reliably detect is $\eta_{p}^{2}$ = .01.

#### Materials and procedure

**Figure 8.**
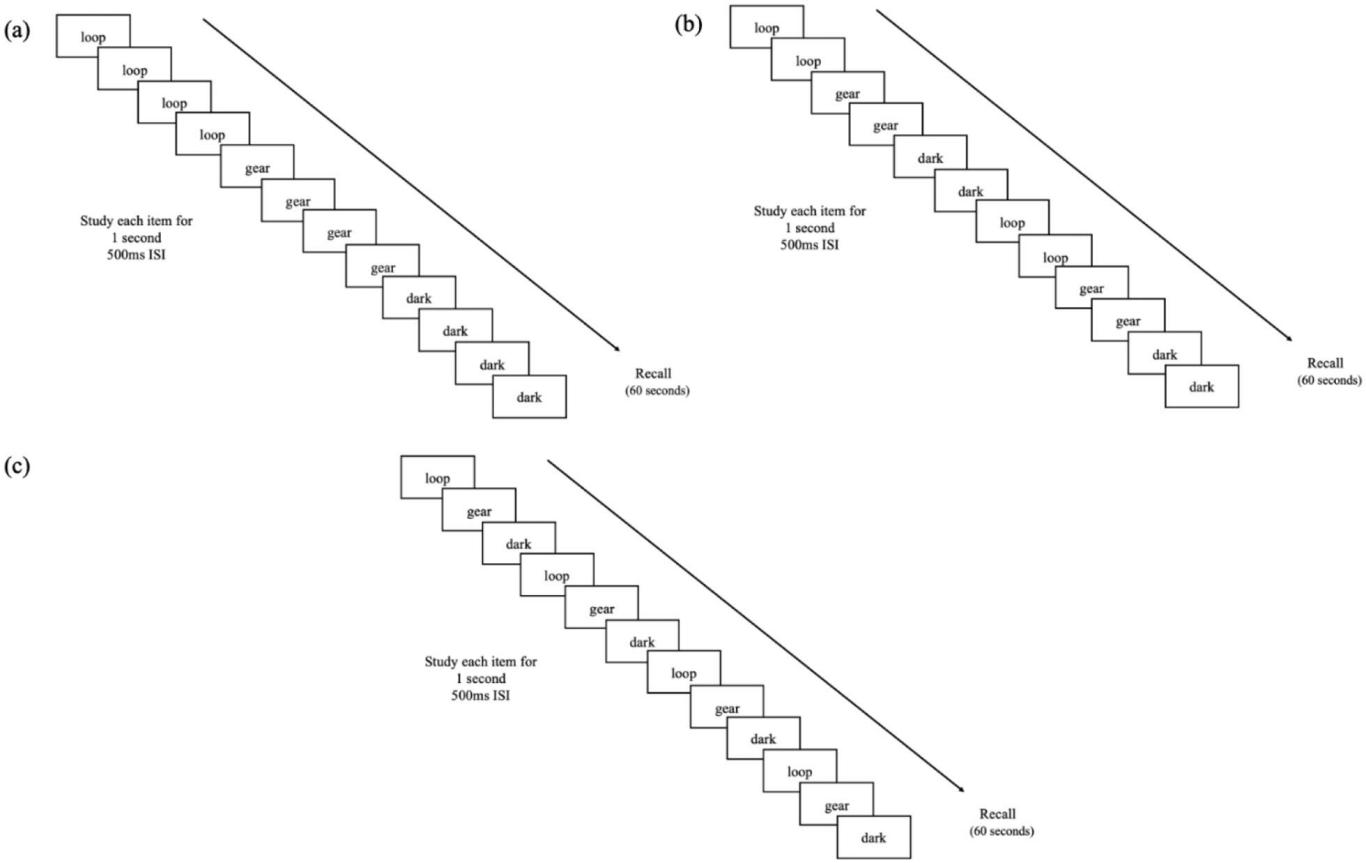
Examples of a list with (a) each word presented four consecutive times for 1 s each, (b) a list with each word presented twice consecutively for 1 s each then again twice consecutively for 1 s each, and (c) a list with each word presented four times for 1 s each (c) in Experiment 2a.

The task in Experiment 2a was similar to the task in Experiment 1a except that each word presentation occurred for just 1 s at a time with a 500-ms inter-stimulus interval (ISI) between every successive presentation of the same word (with the ISI breaking up the total consecutive study time into presentations of 1 s at a time). For example, in the massed condition (i.e., 4 s × 1), a given word appeared for 1 s followed by a 500-ms interval, appeared again for 1 s followed by a 500-ms interval, appeared a third time for 1 s followed by a 500-ms interval, and appeared a final time for 1 s followed by a 500-ms interval before the presentation of the next word which followed the same schedule. Thus, on two of the lists participants studied each word four times in immediate succession for 1 s each, on another two lists participants studied each word two times in immediate succession for 1 s each and then another two times in immediate succession for 1 s each, and on another two lists participants studied each word four times for 1 s each (i.e., 4 s × 1 study schedule: 1-1-1-1; 2 s × 2 study schedule: 1-1 . . . 1-1; the 1 s × 4 study schedule: 1 . . . 1 . . . 1 . . . 1; the “ . . . ” refers to the intervening words when going from one presentation of the list until the next). Examples of the different types of lists are illustrated in Figure 8. Participants again either studied lists containing more concrete words (i.e., easier words to remember; *n* = 48) or less concrete words (i.e., more difficult words to remember; *n* = 48).

### Results

**Figure 9.**
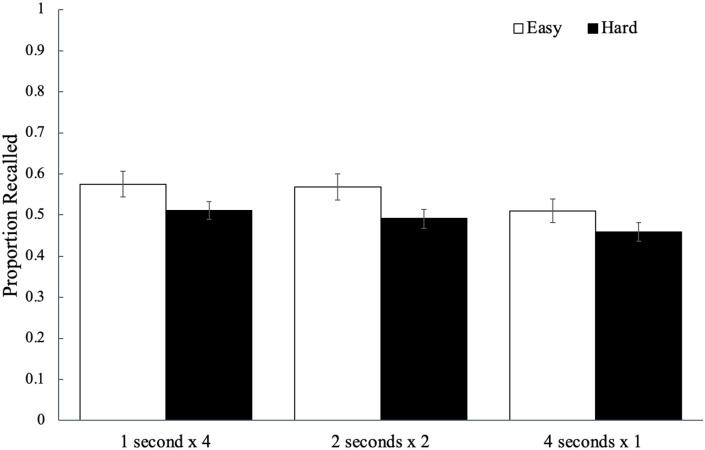
The average proportion of words correctly recalled as a function of word difficulty and how a fixed study time was distributed across presentations of a given word in Experiment 2a. Error bars reflect the standard error of the mean.

Recall performance for each study schedule as a function of word difficulty is shown in Figure 9. A 2 (word difficulty: easy, hard) × 3 (study schedule: 1 s × 4, 2 s × 2, 4 s × 1) mixed ANOVA did not reveal a significant main effect of word difficulty, *F*(1, 94) = 3.43, *p* = .067, $\eta_{p}^{2}$ = .04, BF_01_ = 0.83, such that participants recalled a similar proportion of easy words (*M* = 0.55, *SD* = 0.20) as hard words (*M* = 0.49, *SD* = 0.14). However, there was a main effect of study schedule, *F*(2, 188) = 9.56, *p* < .001, $\eta_{p}^{2}$ = .09, BF_10_ > 100, such that words studied four (distributed) times for 1 s (*M* *=* 0.54, *SD* *=* 0.19) were recalled better than the words studied four times for 1 s in immediate succession (*M* *=* 0.49, *SD* = 0.18, *p*_bonf_ < .001, *d* = 0.43) but not the words studied twice for 1 s and then twice again for 1 s each (*M* = 0.53, *SD* = 0.20, *p*_bonf_ = .961, *d* = 0.10); additionally, recall for the words studied twice for 1 s each, and then again twice for 1 s each was significantly greater than recall for the words studied four times for 1 s in immediate succession (*p*_bonf_ = .005, *d* = 0.33). Word difficulty did not interact with study schedule, *F*(2, 188) = 0.44, *p* = .644, $\eta_{p}^{2}$ = .01, BF_01_ = 9.60.

To examine recall as a function of the number of non-consecutive presentations while controlling for retention interval, we conducted a logistic MLM with item-level recall modelled as a function of number of word presentations and retention interval. Results revealed that, when controlling for retention intervals, the number of non-consecutive presentations significantly predicted recall, e^B^ = 1.07, CI = [1.03, 1.12], *z* = 3.68, *p* < .001, such that distributed practice enhanced memory.

**Figure 10.**
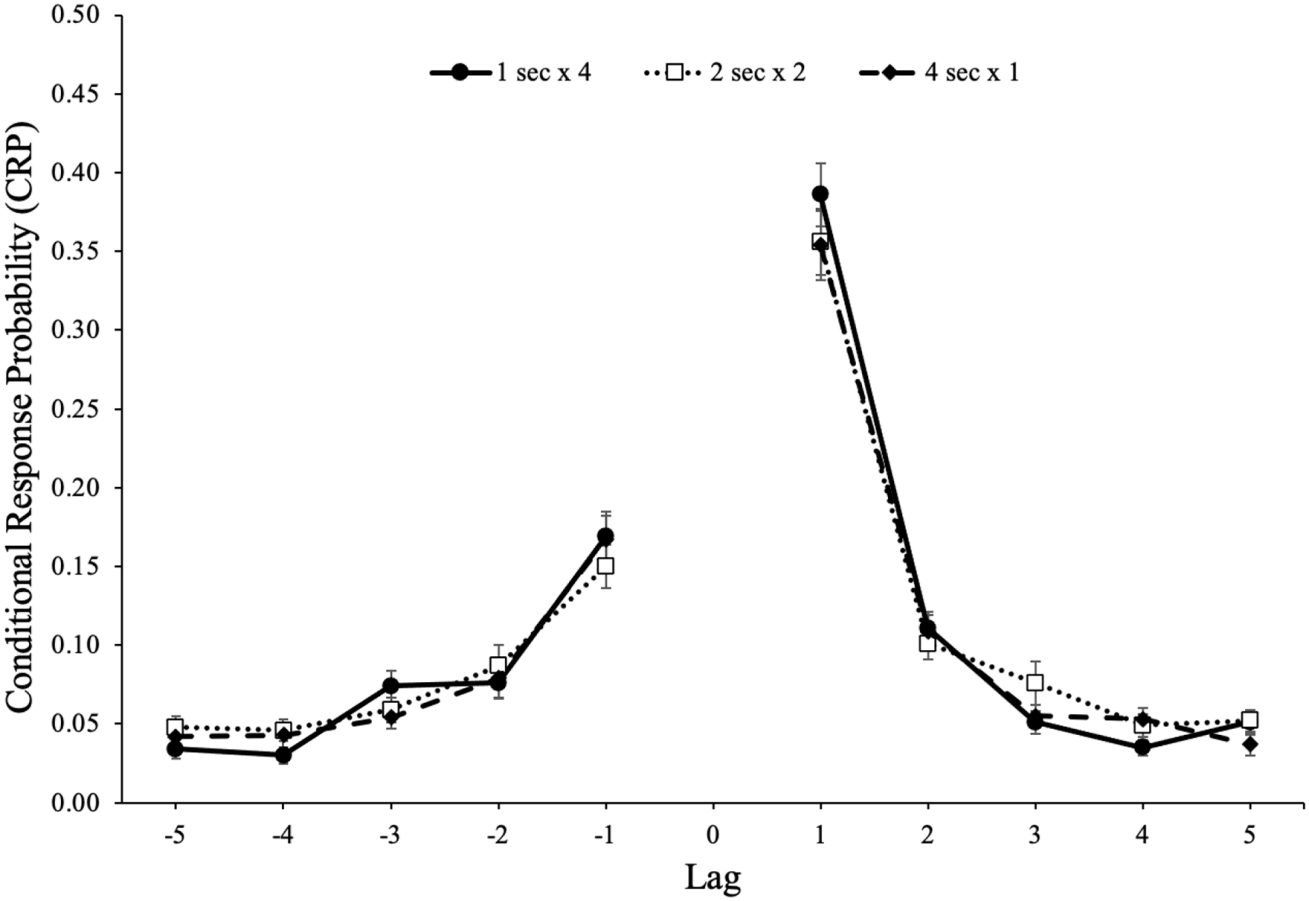
Conditional-response probability (CRP) functions for forward and backward transitions as a function of lag and study schedule in Experiment 2a. Error bars reflect the standard error of the mean.

CRP functions for forward and backward transitions as a function of lag and study schedule are shown in Figure 10. A 5 (lag: 1–5) × 2 (direction: forward vs backward) × 2 (study schedule: 1 s × 4, 2 s × 2, 4 s × 1) repeated-measures ANOVA revealed a forward preference for the direction of transitions, *F*(1, 95) = 89.19, *p* < .001, $\eta_{p}^{2}$ = .48, BF_10_ > 100, and strong adjacency effects, *F*(4, 380) = 285.31, *p* < .001, $\eta_{p}^{2}$ = .75, BF_10_ > 100. In addition, lag significantly interacted with direction, *F*(4, 380) = 62.96, *p* < .001, $\eta_{p}^{2}$ = .40, BF_10_ > 100, such that participants demonstrated a stronger preference for words in the forward direction of 1 lag. However, there was not a significant main effect of study schedule, *F*(2, 190) = 0.48, *p* = .622, $\eta_{p}^{2}$ = .01, BF_01_ > 100. Moreover, study schedule did not interact with direction, *F*(2, 190) = 0.22, *p* = .806, $\eta_{p}^{2}$ < .01, BF_01_ > 100, or lag, *F*(8, 760) = 1.45, *p* = .172, $\eta_{p}^{2}$ = .02, BF_01_ > 100, and there was not a three-way interaction between direction, lag, and study schedule, *F*(8, 760) = 1.03, *p* = .414, $\eta_{p}^{2}$ = .01, BF_01_ > 100.

**Figure 11.**
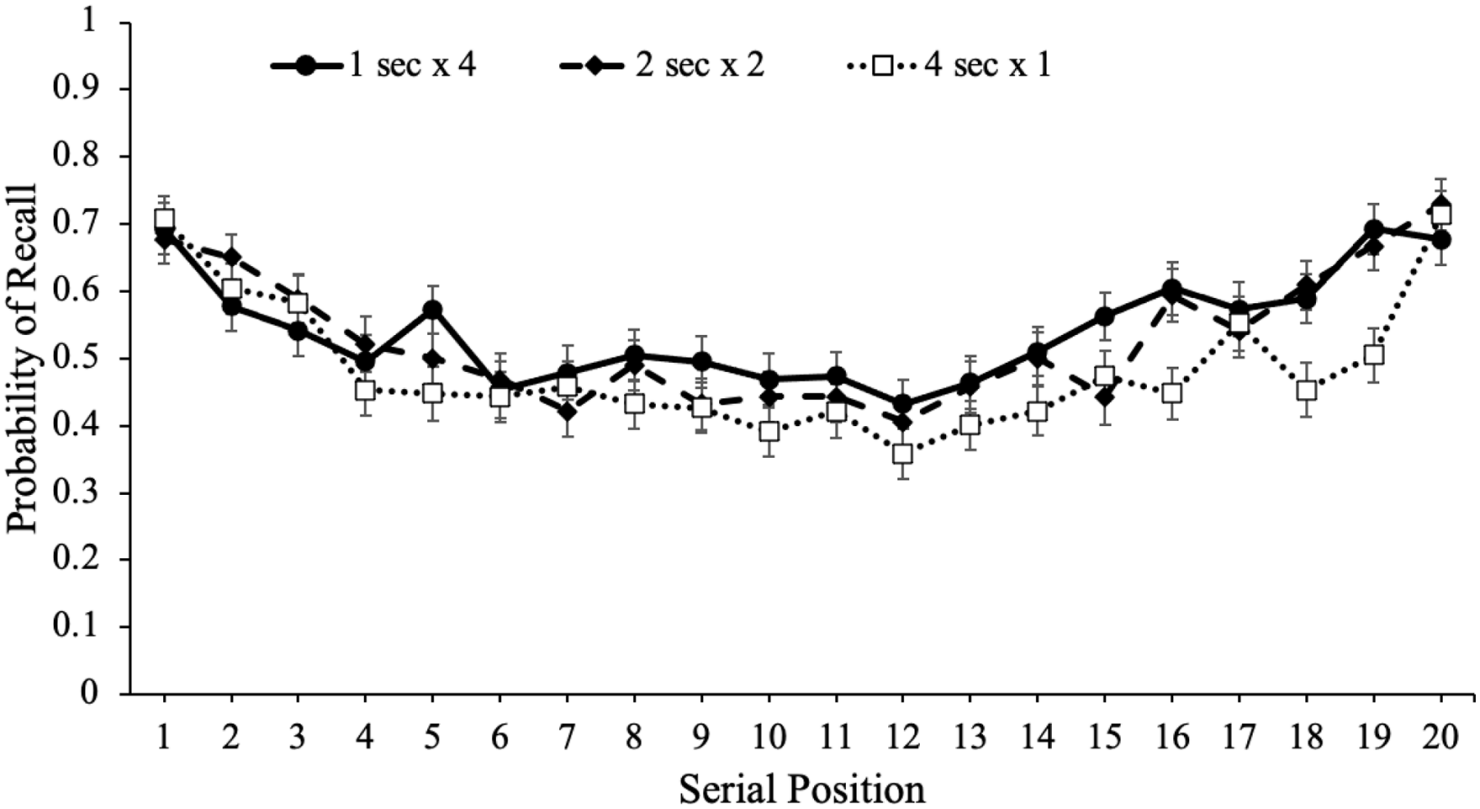
Free recall probability as a function of study schedule and serial position in Experiment 2a. Error bars reflect the standard error of the mean.

Next, we again investigated serial position effects for each study schedule, which are shown in Figure 11. A logistic MLM with item-level recall modelled as a function of serial position and number of non-consecutive presentations revealed that serial position did not significantly predict recall, e^B^ = 1.01, CI = [1.00, 1.01], *z* = 1.63, *p* = .102. However, number of non-consecutive presentations significantly predicted recall, e^B^ = 1.08, CI = [1.05, 1.12], *z* = 5.00, *p* < .001, and serial position interacted with number of non-consecutive presentations, e^B^ = 1.01, CI = [1.00, 1.01], *z* = 2.64, *p* = .008, such that serial position was a stronger predictor of recall when participants were presented with items for four times for 1 s consecutively or twice for 1 s and then twice again for 1 s.

To supplement these findings, we again computed quadratic regressions with serial position predicting recall for each study schedule. As shown in Table 3, quadratic models significantly predicted recall such that there were serial position effects in participants’ recall whereby primacy items were recalled better than items in the middle and at the end of the list. Similar to Experiment 1, these models also demonstrated that when participants were presented with each word four times (but spaced), the serial position curve flattened.

**Table 3.** Quadratic regressions with recall predicted by serial position for each study schedule in Experiment 2a.

Study schedule	*R*2	*b*1	*b*2	*F*	*p*
1 s × 4	.020	−.044	.002	38.48	<.001
2 s × 2	.033	−.062	.003	65.90	<.001
4 s × 1	.029	−.062	.003	58.26	<.001

### Discussion

Consistent with Experiment 1, distributed practice resulted in better memory performance than studying the words a single time (massed). Specifically, even when each word was only presented for 1 s at a time (i.e., controlling for the number of presentations such that all massed and spaced lists had four presentations for each item), spacing the repeated study opportunities enhanced memory performance. Thus, even when controlling for the number of presentations, spaced repetitions of a word resulted in better memory than repetitions of a word that occurred in succession, indicating that the spacing effect may occur even in single encoding sessions. However, we again wanted to replicate these findings when the recall test follows a distracting delay.

## Experiment 2b

In Experiment 2a, we largely replicated the effects of Experiment 1a such that distributed practice led to better recall, even if a given word only appeared for 1 s at a time with a 500-ms interval between every successive presentation of a word. In Experiment 2b, we again compared a massed equivalent of the spaced repetition study schedules to allow for a direct comparison of spacing versus repetition. We aimed to replicate the effects of Experiment 1b such that spaced repetitions of to-be-remembered words can enhance memory even when final recall is delayed.

### Method

#### Participants

After exclusions, participants were 96 undergraduate students (*M_age_* = 20.02, *SD_age_* = 1.76; 90 female, 6 male; 39 Asian/Pacific Islander, 4 Black, 12 Hispanic, 29 White, 12 other/unknown) recruited from the UCLA Human Subjects Pool. Participants were tested online and received course credit for their participation. Participants were excluded from analysis if they admitted to cheating (e.g., writing down answers) in a post-task questionnaire (they were told they would still receive credit if they cheated). This exclusion process resulted in one exclusion. A sensitivity analysis based on the obtained sample indicated that for a 2 (word difficulty: easy, hard) × 3 (study schedule: 1 s × 4, 2 s × 2, 4 s × 1) mixed ANOVA, assuming alpha = .05, power = .80, and a high correlation (*r* = .74) between repeated measures, the smallest effect (recall as a function of study schedule) the design could reliably detect is $\eta_{p}^{2}$ = .01.

#### Materials and procedure

The task in Experiment 2b was similar to the task in Experiment 2a except that instead of completing each free recall test immediately after the study phase, participants first completed the 30-s distraction task used in Experiment 1b. Again, participants either studied lists containing more concrete words (i.e., easier words to remember; *n* = 48) or less concrete words (i.e., more difficult words to remember; *n* = 48).

### Results

**Figure 12.**
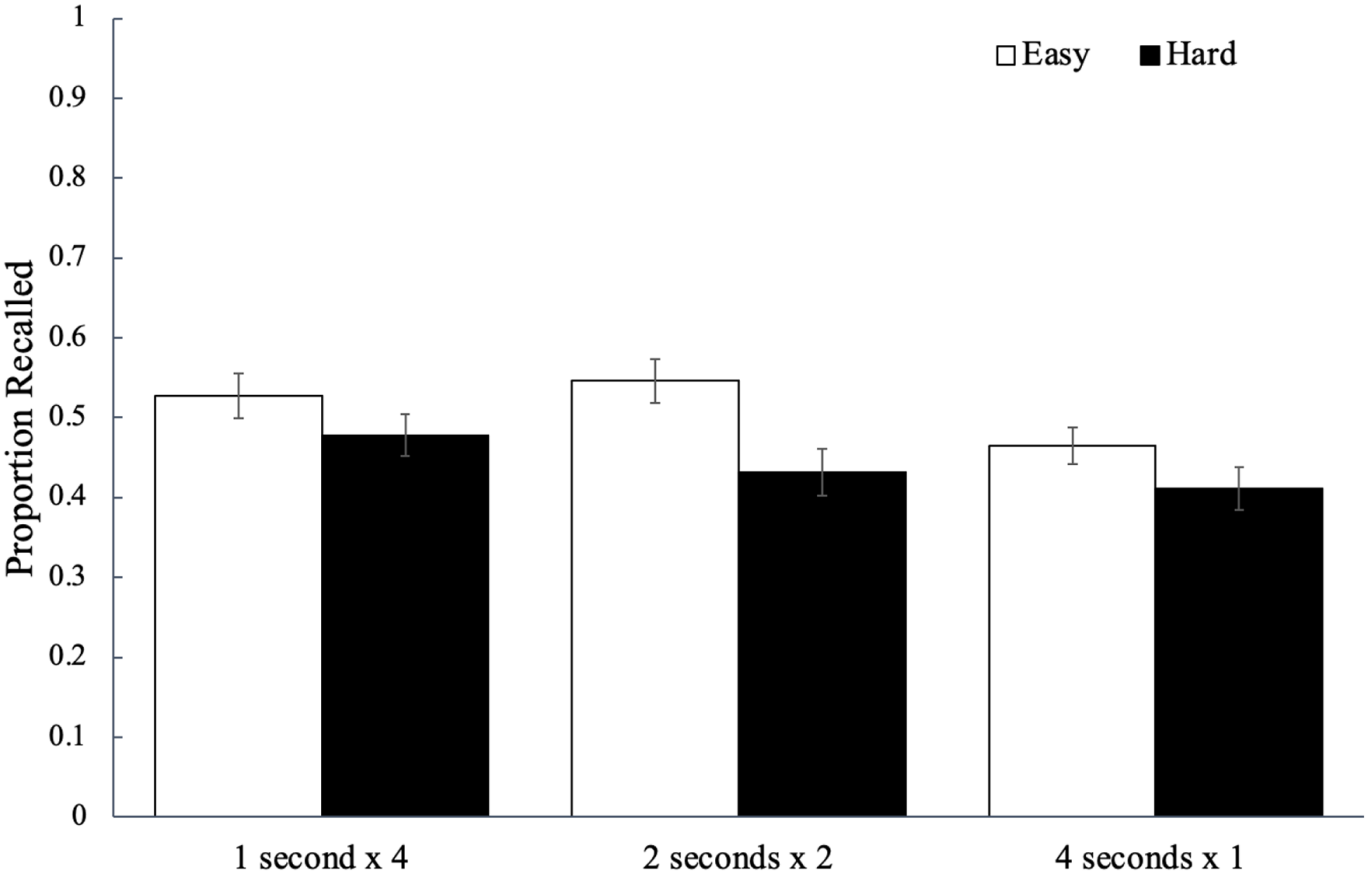
The average proportion of words correctly recalled as a function of word difficulty and how a fixed study time was distributed across presentations of a given word in Experiment 2b. Error bars reflect the standard error of the mean.

Recall performance for each study schedule as a function of word difficulty is shown in Figure 12. A 2 (word difficulty: easy, hard) × 3 (study schedule: 1 s × 4, 2 s × 2, 4 s × 1) mixed ANOVA revealed a main effect of word difficulty, *F*(1, 94) = 4.55, *p* = .036, $\eta_{p}^{2}$ = .05, BF_10_ = 1.83, such that participants recalled more easy words (*M* = 0.51, *SD* = 0.16) than hard words (*M* = 0.44, *SD* = 0.18). Furthermore, there was a main effect of study schedule, *F*(2, 188) = 12.18, *p* < .001, $\eta_{p}^{2}$ = .12, BF_10_ > 100, such that words studied four (distributed) times for 1 s (*M* *=* 0.50, *SD* *=* 0.19) were recalled better than words studied four consecutive times for 1 s (*M* *=* 0.44, *SD* = 0.17, *p*_bonf_ < .001, *d* = 0.48) but not the words studied twice for 1 s and then twice again for 1 s each (*M* = 0.49, *SD* = 0.20, *p*_bonf_ > .999, *d* = 0.10); additionally, recall for the words twice for 1 s and then twice again for 1 s each was significantly greater than recall for the words studied four consecutive times for 1 s (*p*_bonf_ < .001, *d* = 0.38). However, word difficulty interacted with study schedule, *F*(2, 188) = 3.54, *p* = .031, $\eta_{p}^{2}$ = .04, BF_10_ = 1.35, such that when studying each word twice for 1 s and then twice again for 1 s each, participants recalled easier words better than harder words (*p*_bonf_ = .041).

To examine recall as a function of the number of non-consecutive presentations while controlling for retention interval, we conducted a logistic MLM with item-level recall modelled as a function of number of word presentations and retention interval. Results revealed that, when controlling for retention intervals, the number of non-consecutive presentations significantly predicted recall, e^B^ = 1.25, CI = [1.21, 1.30], *z* = 11.49, *p* < .001, such that distributed practice enhanced memory.

**Figure 13.**
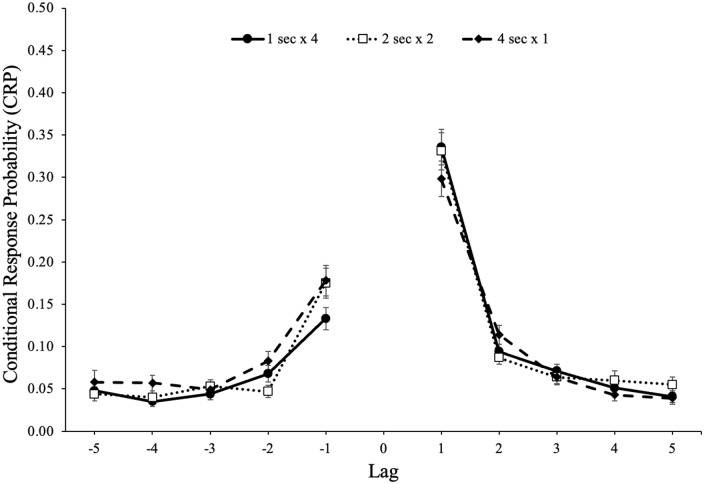
Conditional-response probability (CRP) functions for forward and backward transitions as a function of lag and study schedule in Experiment 2b. Error bars reflect the standard error of the mean.

CRP functions for forward and backward transitions as a function of lag and study schedule are shown in Figure 13. A 5 (lag: 1–5) × 2 (direction: forward vs backward) × 2 (study schedule: 1 s × 4, 2 s × 2, 4 s × 1) repeated-measures ANOVA revealed a forward preference for the direction of transitions, *F*(1, 95) = 85.40, *p* < .001, $\eta_{p}^{2}$ = .47, BF_10_ > 100, and strong adjacency effects, *F*(4, 380) = 247.76, *p* < .001, $\eta_{p}^{2}$ = .72, BF_10_ > 100. In addition, lag significantly interacted with direction, *F*(4, 380) = 37.25, *p* < .001, $\eta_{p}^{2}$ = .28, BF_10_ > 100, such that participants demonstrated a stronger preference for words in the forward direction of 1 lag. However, there was not a significant main effect of study schedule, *F*(2, 190) = 1.43, *p* = .243, $\eta_{p}^{2}$ = .02, BF_01_ > 100. Moreover, study schedule interacted with direction, *F*(2, 190) = 4.74, *p* = .010, $\eta_{p}^{2}$ = .05, BF_10_ = 0.08, such that participants studying each word four (distributed) times for 1 s showed weaker lag-recency effects than participants studying each word four consecutive times for 1 s (*p*_bonf_ = .014), but there was not an interaction between study schedule and lag, *F*(8, 760) = 1.25, *p* = .268, $\eta_{p}^{2}$ = .01, BF_01_ > 100. Furthermore, there was not a three-way interaction between direction, lag, and study schedule, *F*(8, 760) = 1.49, *p* = .157, $\eta_{p}^{2}$ = .02, BF_01_ > 100.

**Figure 14.**
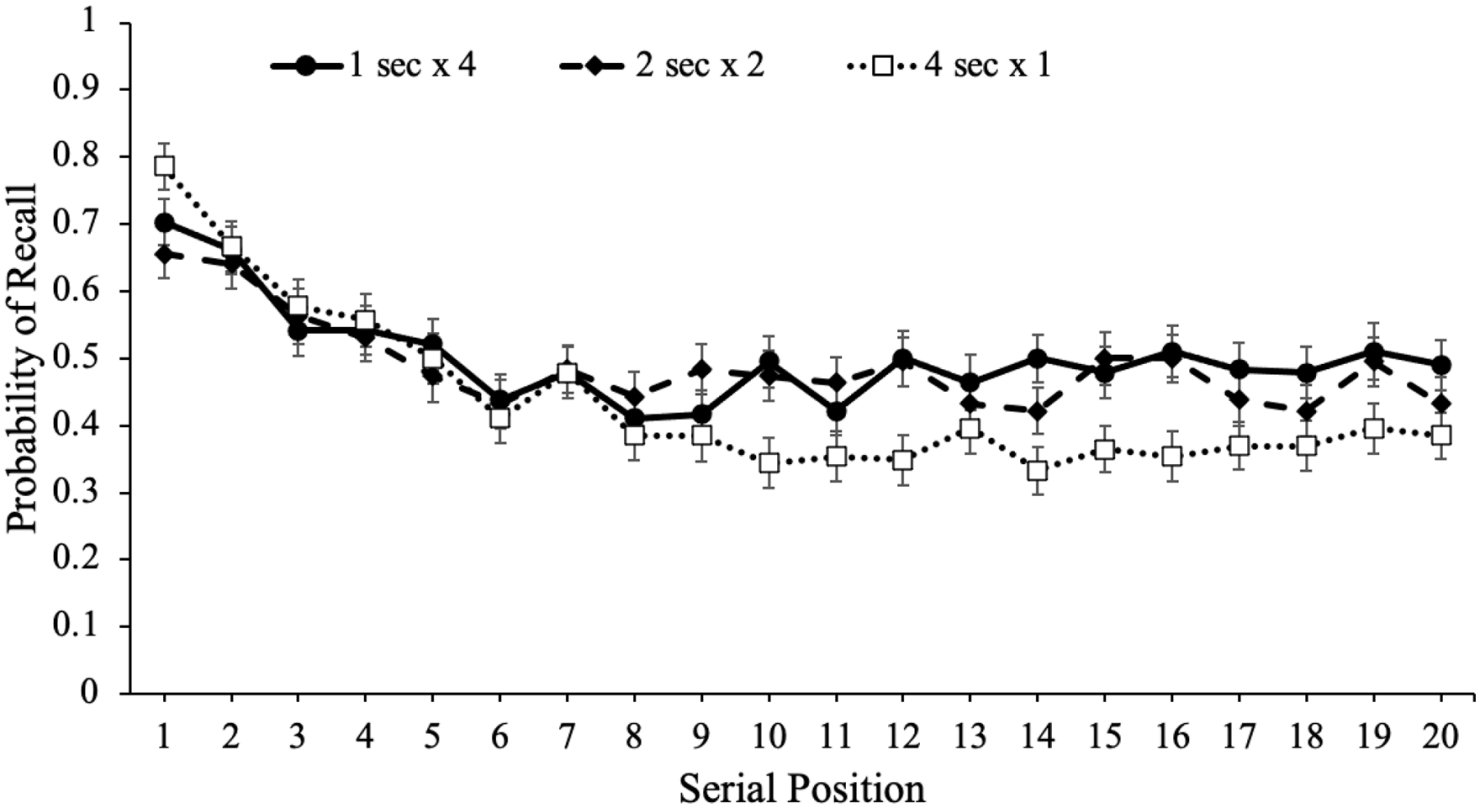
Free recall probability as a function of study schedule and serial position in Experiment 2b. Error bars reflect the standard error of the mean.

Next, we again investigated serial position effects for each study schedule, which are shown in Figure 14. A logistic MLM with item-level recall modelled as a function of serial position and number of non-consecutive presentations revealed that serial position significantly predicted recall, e^B^ = 0.96, CI = [0.95, 0.96], *z* = –12.49, *p* < .001, such that primacy items were better recalled than middle and recency items. In addition, number of non-consecutive presentations significantly predicted recall, e^B^ = 1.09, CI = [1.06, 1.13], *z* = 5.55, *p* < .001, and serial position interacted with number of non-consecutive presentations, e^B^ = 1.01, CI = [1.01, 1.02], *z* = 5.02, *p* < .001, such that serial position was a stronger predictor of recall when study time was not distributed.

To supplement these findings, we again computed quadratic regressions with serial position predicting recall for each study schedule. As shown in Table 4, quadratic models significantly predicted recall such that there were serial position effects in participants’ recall whereby primacy items were recalled better than items in the middle and at the end of the list. However, these models also demonstrated that when study time was distributed, the serial position curve flattened.

**Table 4.** Quadratic regressions with recall predicted by serial position for each study schedule in Experiment 2b.

Study schedule	*R*2	*b*1	*b*2	*F*	*p*
1 s × 4	.013	−.040	.002	26.19	<.001
2 s × 2	.011	−.030	.001	21.28	<.001
4 s × 1	.053	−.066	.002	108.39	<.001

### Discussion

In Experiment 2b, results largely replicated the effects of Experiment 1 such that spaced repetitions of to-be-remembered words enhanced recall. However, in both Experiments 1 and 2, words were always presented in the same fixed order for each study schedule, making it unclear whether this same pattern would occur if the words were presented in random order across repetitions of the same list. This possibility was explored in Experiment 3.

## Experiment 3

In Experiment 3, participants completed a similar task as in Experiment 1b but with words presented in random order. Similar to Experiments 1 and 2, we expected multiple study opportunities to improve memory performance regardless of the fewer temporal-contextual cues to aid in the recall of words and regardless of the difficulty of the to-be-remembered words.

### Method

#### Participants

After exclusions, participants were 110 undergraduate students (*M_age_* = 20.41, *SD_age_* = 1.48; 78 female, 31 male, 1 other; 49 Asian/Pacific Islander, 2 Black, 25 Hispanic, 26 White, 8 other/unknown) recruited from the UCLA Human Subjects Pool. Participants were tested online and received course credit for their participation. Participants were excluded from analysis if they admitted to cheating (e.g., writing down answers) in a post-task questionnaire (they were told they would still receive credit if they cheated). This exclusion process resulted in three exclusions. A sensitivity analysis indicated that for a 2 (word difficulty: easy, hard) × 3 (study schedule: 1 s × 4, 2 s × 2, 4 s × 1) mixed ANOVA, assuming alpha = .05, power = .80, and a high correlation (*r* = .66) between repeated measures, the smallest effect (recall as a function of study schedule) the design could reliably detect is $\eta_{p}^{2}$ = .01.

#### Materials and procedure

The task in Experiment 3 was similar to the task in Experiment 1b except that in the study schedules where participants viewed the words more than once, the words were presented in random order rather than appearing in the same order across the successive presentations on the same list. Participants again either studied lists containing more concrete words (i.e., easier words to remember; *n* = 55) or less concrete words (i.e., more difficult words to remember; *n* = 55). Finally, after the task, participants reported which of the three study schedules they preferred.

### Results^5^

**Figure 15.**
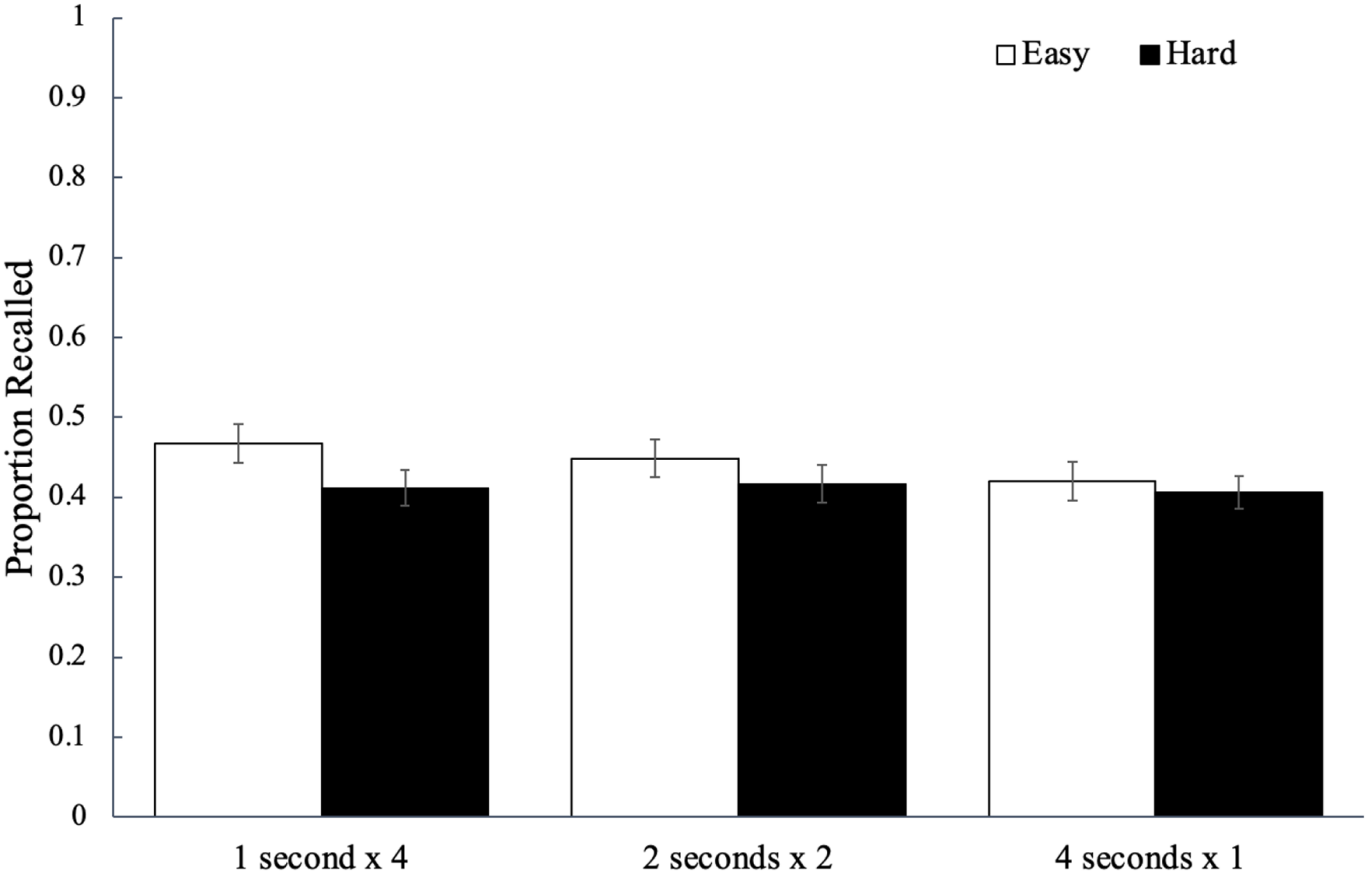
The average proportion of words correctly recalled as a function of word difficulty and how a fixed study time was distributed across presentations of a given word in Experiment 3. Error bars reflect the standard error of the mean.

Recall performance for each study schedule as a function of word difficulty is shown in Figure 15. A 2 (word difficulty: easy, hard) × 3 (study schedule: 1 s × 4, 2 s × 2, 4 s × 1) mixed ANOVA did not reveal a significant main effect of word difficulty, *F*(1, 108) = 1.37, *p* = .244, $\eta_{p}^{2}$ = .01, BF_01_ = 2.01, such that participants recalled a similar proportion of easy words (*M* = 0.45, *SD* = 0.16) as hard words (*M* = 0.41, *SD* = 0.15). There was not a significant main effect of study schedule, *F*(2, 216) = 2.10, *p* = .125, $\eta_{p}^{2}$ = .02, BF_01_ = 4.46, such that words studied four times for 1 s (*M* *=* 0.44, *SD* *=* 0.17), twice for 2 s (*M* *=* 0.43, *SD* = 0.18), and once for 4 s (*M* *=* 0.41, *SD* = 0.17) were similarly recalled. Word difficulty did not interact with study schedule, *F*(2, 216) = 1.19, *p* = .308, $\eta_{p}^{2}$ = .01, BF_01_ = 5.82.

Finally, we examined participants’ study schedule preferences. A chi-square goodness-of-fit test indicated that there were no differences in participants’ study schedule preference, χ^2^(2) = 1.65, *p* = .437, such that a similar proportion of participants preferred the 1 s × 4 schedule (28%), 2 s × 2 schedule (34%), and 4 s × 1 schedule (38%).

### Discussion

Unlike in Experiments 1 and 2, studying an item multiple times (but for shorter durations) in a massed encoding phase (but with words in a random order) did not result in significantly better recall than studying each item a single time for the same cumulative duration. However, the recall patterns observed in Experiment 3 were similar to those in Experiments 1 and 2, indicating that the benefits of repetition may not have been absent but rather reduced when studying words in an unorganised or random fashion. In addition, the different lags between items may have obscured the distributed practice effect observed in Experiments 1 and 2. For example, as a consequence of the random order, some items were repeated after short lags which may confer some benefit, some items were repeated after moderate lags which may confer greater or lesser benefit, and some items were repeated after longer lags which may confer little benefit if study-phase retrieval fails.

From an encoding variability standpoint, having two or four chances to create a mental image, group words together, or generate a sentence involving a given word and the same neighbouring words may be more beneficial than having two or four chances to engage in these elaborative encoding strategies with different sets of neighbouring words. From the standpoint of the study-phase retrieval interpretation of the spacing effect, varying the words that preceded and followed the repetition of a given word may have reduced the frequency with which participants retrieved the prior presentation of a repeated word. Such a possibility is supported by the results of Appleton-Knapp et al. (2005) who had participants study a series of magazine-type advertisements for hypothetical products. After studying the advertisements, participants were tested on their ability to recall the product names when given a cue, such as a tag line for the product. Results revealed that varying the visual features for two advertisements of a given product increased participants’ ability to recall the product name when the advertisements were shown quite close to each other, but impaired recall of the product name when a larger number of advertisements for other products intervened.

### Cross-experiment comparisons

To directly compare recall trends in Experiment 1b and Experiment 3, we conducted a 2 (word order: fixed, random) × (3 study schedule) between-experiment post hoc (after having conducted our initial analyses) ANOVA on recall performance. Results revealed a main effect of study schedule, *F*(2, 398) = 11.45, *p* < .001, $\eta_{p}^{2}$ = .05, BF_10_ > 100, such that spaced repetitions still led to a recall advantage, a main effect of word order, *F*(1, 199) = 4.80, *p* = .030, $\eta_{p}^{2}$ = .02, BF_10_ = 1.83, such that recall was better when the words appeared in the same fixed order, but the interaction between word order and study schedule failed to reach significance, *F*(2, 398) = 2.49, *p* = .084, $\eta_{p}^{2}$ = .01, BF_01_ = 2.50. Thus, the present series of studies generally suggest that multiple study opportunities can enhance memory in a single encoding session, but this effect is greatest when information is studied in the same fixed order.

Moreover, we were also interested in how the ISI (which broke up consecutive study opportunities into presentations of 1 s) impacted performance. To directly compare recall trends in Experiments 1 and 2, we conducted a 2 (ISI: none, 0.5 s between each 1 s presentation) × (3 study schedule) between-experiment post hoc ANOVA on recall performance. Results revealed a main effect of study schedule, *F*(2, 734) = 39.83, *p* < .001, $\eta_{p}^{2}$ = .10, BF_10_ > 100, such that spaced repetitions still led to a recall advantage. However, there was not an effect of having an ISI, *F*(1, 367) < 0.01, *p* = .971, $\eta_{p}^{2}$ < .01, BF_01_ = 5.88, and study schedule did not depend on having an ISI, *F*(2, 734) = 0.04, *p* = .962, $\eta_{p}^{2}$ < .01, BF_01_ = 46.36. Thus, ISIs breaking presentations into 1 s at a time had little effect on memory performance.

## General discussion

Across the five experiments we have reported, several key findings emerged. First, within a single encoding session, when total study time was kept constant, dividing that time into multiple (spaced) exposures of the to-be-learned words enhanced overall learning of those words, even though dividing the time meant reducing the study time available during every presentation of a given item (but this effect was reduced when words were presented in random order). Second, the benefit of the spaced repetition study schedules endured whether the recall test was immediate or followed a delay. Third, even when each word was only presented for 1 s at a time (with a 500-ms interval between every successive presentation of the same word), when study time for a given word was distributed, memory was enhanced. Thus, the present study revealed that the spacing effect, which generally indicates that learners should distribute study time across multiple study sessions (see R. A. Bjork & Allen, 1970; Cepeda et al., 2006; Greene, 2008; Karpicke & Bauernschmidt, 2011), can manifest even within a single encoding session (consistent with prior work, see Delaney et al., 2010; Glenberg, 1976; Maddox, 2016). As such, if students choose to engage in massed studying (say, by virtue of constraints on their study time or a failure to appreciate the benefits of spaced study sessions), then studying the information twice but for half the time may produce memory benefits in a single study session.

The present results seem consistent with the consolidation and study-phase retrieval theories of the spacing effect—that is, multiple study opportunities may have resulted in additional item representations (R. A. Bjork & Allen, 1970) and/or the retrieval of earlier word presentations (Appleton-Knapp et al., 2005; Greene, 1989; Thios & D’Agostino, 1976) leading to enhanced recall. In addition to theories of the spacing effect, the benefits of distributed study time within a given list may be attributable to rehearsal. For example, when studying lists of words, participants generally start with rote rehearsal but switch to more elaborate encoding strategies towards the end of the list (Delaney & Knowles, 2005). In addition, prior work has shown that, when participants are asked to overtly rehearse items, the number of rehearsals increased with increased lag, which corresponded to increased memory performance (Rundus, 1971; see also Verkoeijen & Delaney, 2008; Zimmerman, 1975). Thus, if participants opt to use more effective encoding strategies later in the list and are given multiple opportunities to study the words, more words in the list may benefit from the use of these elaborative encoding strategies when study time is distributed than when words are only studied once but for a longer duration.

In the current study, we observed a flattening of the serial position curve when learners were given multiple (but shorter) opportunities to study each word, potentially reflecting more efficient rehearsal processes (see Murphy et al., 2022a). Specifically, the cumulative rehearsal occurring when only given a single (but longer) opportunity to study each item may be replaced with more effective encoding strategies on later presentations. There may also be retrieval practice benefits (R. A. Bjork, 1988; Roediger & Butler, 2011; see also Balota et al., 2007a) when words are presented multiple times, leading to enhanced recall, particularly for words in the middle of the list (as evidenced by the poorer recall of mid-list words when only studying an item a single time).

In the classroom, the efficacy of spaced learning has been demonstrated extensively (Carpenter et al., 2012; Dempster, 1988; Kapler et al., 2015; Pashler et al., 2007; Sobel et al., 2011) such that students who space their studying demonstrate enhanced performance. Yet, students sometimes do not take advantage of the spacing effect and instead choose to engage in massed studying. When engaging in this less effective technique (in terms of long-term retention), the present study demonstrated that learners’ organisation of their massed practice can still be strategically distributed to optimise memory performance. For example, if the time remaining before a memory test is limited, short but repeated studying may result in better memory performance than studying the same information less frequently but for a longer duration (although there are likely limits to this technique, e.g., studying each item in a list 20 times for 200 ms each may not be advantageous for learning). However, the effect of spacing observed in the present experiments was relatively small, limiting the power to detect potential interactions with item difficulty and the extent to which spacing influences lag-recency effects. Nevertheless, the current study suggests that it may be possible to receive the benefits of spacing without increasing the total interval from beginning the study process to finishing the study process.

To the extent that our findings using word lists generalise to the learning of more complex materials, our results suggest it may be advantageous for students engaging in massed studying to study to-be-learned material covered on an exam multiple times, even if each encoding session is shorter. For example, the present research was motivated in part by the fact that students are now watching prerecorded lectures in most remote courses, and they can watch these videos at various times and variable speeds (i.e., 0.5×, 1×, 1.5×, 2×). Given that flexibility, some students may watch lecture videos a single time at their original speed, while others may watch a given lecture video more than once and perhaps at an increased speed. Thus, two students may spend the same amount of time watching a particular lecture video but differ in how that time is distributed (see Murphy et al., 2022b, for a test of this conjecture).

Although the present results suggest that in massed encoding situations, studying information only once may be an inferior learning strategy compared to studying the same information for half the time before restudying that information for a second time in the same encoding session, there are several limitations to these findings. First, although we equated the nominal presentation time across study schedules (i.e., each word was always studied for a total of 4 s), this procedure may not have necessarily equated encoding time. For example, on lists where participants studied each word once for 4 s, learners may have engaged in different encoding operations during the first 2 s than in the last 2 s. Future work may benefit by using overt rehearsal procedures (see Tan & Ward, 2000; Ward et al., 2003) whereby participants rehearse words aloud to elucidate the types of encoding operations employed by the learner on lists with different study schedules.

Next, consistent with prior work demonstrating a spacing effect when massing and spacing is manipulated across different lists (i.e., pure-lists; e.g., Kahana & Howard, 2005; Toppino & Schneider, 1999; see also Delaney & Knowles, 2005; Delaney & Verkoeijen, 2009; but see Hall, 1992), we used a pure-list design in which study schedules were grouped in separate lists. Future work will benefit from using mixed-list approaches in which different study schedules (e.g., massed and spaced words) occur within the same list together (see Delaney & Verkoeijen, 2009, for mixed-list and pure-list comparisons of the spacing effect). Moreover, future work could examine deficient-processing theories whereby people skip rapidly presented items or pay less attention to short-lag repetitions, the effects of rehearsal borrowing, spacing effect strategies, and various study schedules with different presentation rates.

In addition, study schedules with shorter item presentations but more repetitions may have confounded the number of item presentations and retention interval. For example, participants studying each word once for 4 s have an average retention interval of 38 s (when the recall test is immediate) while participants studying each word four times for 1 s each have an average retention interval of 9.5 s. However, Experiments 1b and 2b incorporated a distractor-filled 30-s delay which should reduce any potential effects of different retention intervals, but future work could examine the effects of a much longer delay (such as 5 min or even 24 hr) to further elucidate the potential effects of different retention intervals using these different study schedules.

In sum, across our five experiments, participants generally demonstrated better recall when the study of to-be-remembered words were distributed, even under the constraint that total study time was kept constant, meaning that each study event—or exposure to a given word—was shorter in duration (consistent with prior work, see Delaney et al., 2010; Glenberg, 1976; Maddox, 2016). Thus, even when engaging in massed study, strategically dividing one’s study time to allow for multiple encoding opportunities, rather than a single encoding event, can lead to enhanced memory performance. However, although there was an advantage of spacing, but not between items presented twice and four times, this suggests that while there are advantages to distributed practice, there may be limits to those benefits. Specifically, spacing can be beneficial compared with massing but the degree to which a learner distributes practice may not necessarily equate to a corresponding benefit to memory performance.

## Concluding comments

Knowing how to learn effectively on one’s own outside the classroom is the “ultimate survival tool” (R. A. Bjork et al., 2013), especially in this era when there is ever more to learn and the requirement to do so falls ever more into our own hands—not only after one’s formal schooling has concluded but during one’s formal schooling as well. We have focused on only one aspect of the multitude of considerations that go into managing one’s learning—how one’s study time is distributed—but it is a crucial aspect.

## Notes

1 As we failed to control for frequency, word frequency effects (see Popov & Reder, in press, for a review of the effects of word frequency on memory) may have reduced the strong effects of concreteness commonly seen in the literature.

2 Given that there is usually a recall advantage for concrete versus abstract words in the literature, we further examined how concreteness affected recall using concreteness as a continuous variable. A logistic MLM with the data treated as hierarchical or clustered (i.e., multilevel) and words nested within individual participants revealed that, when controlling for frequency, concreteness significantly predicted recall (*p* < .001). A similar analysis of the data from Experiment 1b (*p* < .001), Experiment 2a (*p* = .008), Experiment 2b (*p* = .001), and Experiment 3 (*p* < .001) further suggests that the effect of word frequency may have confounded the effect of concreteness when analysed as a categorical variable.

3 For simplicity of the analyses, we did not include word difficulty in our examination of lag-recency effects.

4 As each model was based on different data (i.e., lists with different study schedules), we are unable to compute a formal comparison between models. Rather, we examine the serial position curves in terms of their relative effect sizes (i.e., *R*^2^). Specifically, we frame our discussion in terms of how much of the variance the relative quadratic models accounted for with models accounting for more variance (revealing a greater quadratic trend) indicating more pronounced serial position effects and models accounting for less variance (revealing a weaker quadratic trend) indicating bowed serial position curves.

5 We did not examine conditional-response probabilities (CRPs) or serial position effects in Experiment 3 because words were presented in random order (i.e., a given word would have multiple different neighbours for each lag and multiple serial positions within the list).
